# Mesenchymal stromal cell-associated migrasomes: a new source of chemoattractant for cells of hematopoietic origin

**DOI:** 10.1186/s12964-022-01028-6

**Published:** 2023-02-14

**Authors:** Ilker A. Deniz, Jana Karbanová, Manja Wobus, Martin Bornhäuser, Pauline Wimberger, Jan Dominik Kuhlmann, Denis Corbeil

**Affiliations:** 1 Biotechnology Center (BIOTEC) and Center for Molecular and Cellular Bioengineering, Technische Universität Dresden, 01307 Dresden, Germany; 2 Department of Internal Medicine I, University Hospital Carl Gustav Carus, Technische Universität Dresden, 01307 Dresden, Germany; 3 Center for Regenerative Therapies Dresden, Technische Universität Dresden, 01307 Dresden, Germany; 4 National Center for Tumor Diseases (NCT) & German Cancer Research Center (DKFZ) & Faculty of Medicine, University Hospital Carl Gustav Carus & Technische Universität Dresden & Helmholtz-Zentrum Dresden-Rossendorf (HZDR), Dresden and Heidelberg, Germany; 5 Department of Gynecology and Obstetrics, Medical Faculty and University Hospital Carl Gustav Carus, Technische Universität Dresden, 01307 Dresden, Germany; 6 German Cancer Consortium (DKTK), Partner Site Dresden and German Cancer Research Center (DKFZ), 69120 Heidelberg, Germany; 7 Tissue Engineering Laboratories, Biotechnology Center, Technische Universität Dresden, Tatzberg 47-49, 01307 Dresden, Germany

**Keywords:** Cellular adhesion, Extracellular vesicle, Hematopoietic stem cell, Intercellular signaling, Mesenchymal stromal cell, Migrasome, Motility

## Abstract

**Background:**

Multipotent mesenchymal stromal cells (MSCs) are precursors of various cell types. Through soluble factors, direct cell–cell interactions and other intercellular communication mechanisms such as extracellular vesicles and tunneling nanotubes, MSCs support tissue homeostasis. In the bone marrow microenvironment, they promote hematopoiesis. The interaction between MSCs and cancer cells enhances the cancer and metastatic potential. Here, we have demonstrated that plastic-adherent MSCs isolated from human bone marrow generate migrasomes, a newly discovered organelle playing a role in intercellular communication.

**Results:**

Migrasomes are forming a network with retraction fibers behind the migrating MSCs or surrounding them after membrane retraction. The MSC markers, CD44, CD73, CD90, CD105 and CD166 are present on the migrasome network, the latter being specific to migrasomes. Some migrasomes harbor the late endosomal GTPase Rab7 and exosomal marker CD63 indicating the presence of multivesicular bodies. Stromal cell-derived factor 1 (SDF-1) was detected in migrasomes, suggesting that they play a chemoattractant role. Co-cultures with KG-1a leukemic cells or primary CD34^+^ hematopoietic progenitors revealed that MSC-associated migrasomes attracted them, a process intercepted by the addition of AMD3100, a specific CXCR4 receptor inhibitor, or recombinant SDF-1. An antibody directed against CD166 reduced the association of hematopoietic cells and MSC-associated migrasomes. In contrast to primary CD34^+^ progenitors, leukemic cells can take up migrasomes.

**Conclusion:**

Overall, we described a novel mechanism used by MSCs to communicate with cells of hematopoietic origin and further studies are needed to decipher all biological aspects of migrasomes in the healthy and transformed bone marrow microenvironment.

**Video Abstract**

**Supplementary Information:**

The online version contains supplementary material available at 10.1186/s12964-022-01028-6.

## Background

Mesenchymal stromal cells (MSCs) are multipotent cells that possess unique properties, including the ability to regulate adaptive immune responses through various pathways and to promote hematopoiesis [1–3]. After isolation, based on their adhesion to plastic culture dishes and their proliferation [4], they appear as a heterologous population with the ability to differentiate into osteoblasts, chondrocytes and adipocytes [5, 6]. Thus, they have a great potential for therapeutic applications in regenerative medicine [7, 8].

MSCs are essential components of the bone marrow stem cell niche, where they control hematopoietic stem cell homeostasis [9–13]. MSCs support the quiescence, proliferation, and differentiation of hematopoietic stem and progenitor cells (HSPCs) through direct cell–cell interaction [14–18]. They can also exchange biological information via thin, F-actin-based plasma membrane protrusions referred to as tunneling nanotubes [19–21]. In addition to the direct interaction between MSCs and HSPCs, their communication can be regulated by soluble factors and/or small extracellular membrane vesicles such as exosomes or ectosomes/microvesicles [22–24], further increasing the complexity of molecular mechanisms regulating their close interplay. This intercellular communication through juxtacrine and paracrine actions can also occur in hematologic malignancies and in bone marrow metastases of patients with breast and prostate carcinomas, among others [25–27]. Thus, to intercept these cross-cellular exchanges, it is important to decipher all the mechanisms that regulate them.

In recent years, a novel mechanism of intercellular communication based on large (up to 3 μm) pomegranate-shaped structures encapsulating numerous smaller vesicles, known as migrasomes, has been described [28]. These unique structures develop on the retraction fibers at the rear of migrating cells forming a network of migrasomes [29]. They are enriched in certain tetraspanin membrane proteins [e.g., tetraspanin 4 (TSPAN4)], which regulate their formation [30]. It is proposed that migrasomes play a role as signaling organelles that provide specific biochemical information to neighboring cells [31].

Do MSCs use these specific organelles to exchange information between cellular components of the bone marrow microenvironment? If so, this would add a new facet to the intercellular communication observed in this niche. Using plastic-adherent primary human MSCs derived from bone marrow, we investigated here whether these stromal cells produce such functional entities, and how, if present, they may impact hematopoietic cells. Our data showed that MSCs produced migrasomes and their characterization revealed that they contain MSC markers notably activated leukocyte cell adhesion molecule (ALCAM, CD166) [32, 33], on their surface, while a fraction of them harbors late endosomes/multivesicular bodies (LE/MVB), as suggested by the presence of the small GTPase Rab7 [34], and CD63, a marker of intralumenal vesicles, i.e. precursors of exosomes, associated with LE/MVB [35]. Furthermore, the chemokine stromal cell-derived factor 1 (SDF-1), often referred to as C-X-C motif chemokine 12 (CXCL12), was detected in migrasomes, supporting a possible chemoattractant role [36]. The latter was confirmed by co-culturing MSCs with KG-1a cells, an acute myelogenous leukemia cell line, and primary CD34^+^ HSPCs, where hematopoietic cells were attracted to MSC-associated migrasomes through the CXCR4–SDF-1 axis and were retained there by a CD166-dependent mechanism. Collectively, the finding that MSCs generates migrasomes is important because these organelle-like structures associated with retraction fibers or those that detach from this network after fiber degradation could create a cell-free microenvironment in the bone marrow, while retaining certain cellular components.

## Methods

### Antibodies and reagents

Primary and secondary antibodies used in this study are presented in Additional file 1: Tables S1 and S2, respectively. Recombinant human SDF-1 protein and AMD3100 (Plerixafor) were obtained from Abcam (catalog number (#) ab9798 and #ab120718, respectively, Cambridge, UK). SDF-1 was resuspended in 0.1% bovine serum albumin (BSA) solution at a final concentration of 10 ng/µL, while AMD3100 was resuspended in distilled water at 1 mM. Carbonyl cyanide m-chlorophenyl hydrazine (CCCP) obtained from Sigma-Aldrich (#C2759, St. Louis, MO) was dissolved in dimethyl sulfoxide (DMSO) at a final concentration of 1 mM. Alexa Fluor®488-conjugated Phalloidin (#A12379), MitoTracker™ Red CMXRox (#M75512) and MitoSOX Red (#M36008) were purchased from Thermo Fisher Scientific (Waltham, MA). Human fibronectin (#356008) was purchased from Corning Inc. (Corning, NY) and was dissolved in distilled water at a final concentration of 50 µg/mL. Collagen IV (#C5533), laminin from Engelbreth-Holm-Swarm (EHS) murine sarcoma basement membrane (#L2020), ECMatrix-411™ E8 Laminin Substrate (#CC162-350UG) and poly-l-lysine (PLL) (#P4707) were all obtained from Sigma-Aldrich. Human recombinant laminin 511 (#LN511-0502) and 521 (#LN521-05) were purchased from BioLamina (Sundbyberg, Sweden). Laminins were used as recommended by manufacturers, while collagen IV was dissolved in 0.25% acetic acid solution at a concentration of 1 mg/mL.

### MSCs and CD34^+^ HSPCs isolation and cell culture

#### MSCs

Primary MSCs were extracted from bone marrow aspirates collected from healthy donors who provided informed consent. The study was approved by the local ethics committee (Ethikkommission an der Technischen Universität Dresden [TUD], Ethic board no. EK263122004). The age of the donors (*n* = 4) ranged from 25 to 35 years as previously described [6]. Plastic-adherent MSCs were isolated and cultured in MSC medium (low-glucose DMEM, #31885-023, Thermo Fisher Scientific) and 10% fetal calf serum (FCS; PAA, GE Healthcare) [6, 14] and used between passages 3 and 5. They were cultured on either on human fibronectin (1 or 5 µg/cm^2^), collagen IV (10 µg/cm^2^), various laminin types (5 µg/cm^2^) or PLL (5 µg/cm^2^) coated glass-bottom 35-mm dishes (#P35G-1.5-14 C, Mattek Inc.). Alternatively, they were grown on uncoated glass-bottom 35-mm dishes.

For mitochondrial stress treatment, MSCs growing on fibronectin-coated coverslips for 24 h were incubated with or without 1 µM CCCP for 8 h followed by incubation with 100 nM MitoTracker Red or 5 µM MitoSOX Red fluorogenic dye for 15 min. CD166 blocking assays were done by pre-incubating MSCs with anti-CD166 antibodies (Additional file 1: Table S1, 5 µg/mL) diluted in complete medium for 2 h, followed by the removal of antibody solution and addition of KG-1a cells or CD34^+^ HSPCs for downstream experiments. For control, an IgG1 isotype control antibody was used instead of anti-CD166 antibodies.

#### CD34^+^ HSPCs

The mobilized peripheral blood was collected from four healthy donors, after informed consent and approval by the local ethics committee (Ethikkommission an der TUD, Ethic board no. EK201092004). Mobilization was achieved by subcutaneous injection of granulocyte colony-stimulating factor (7.5 µg/kg per day; Granocyte, Chugai Pharma) [14]. CD34^+^ HSPCs were isolated directly after leukapheresis by magnetic-activated cell sorting (MACS) (#130-046-702, Miltenyi Biotec, Bergisch Gladbach, Germany) technology based on CD34, as we described previously [14, 20, 37]. CD34^+^ HSPCs were cultured in serum-free HSPC medium (CellGenix® GMP SCGM, CellGenix GmbH, Freiburg, Germany) supplemented with early acting cytokines (50 ng/mL stem cell factor, (CellGenix), 50 ng/mL fms-related tyrosine kinase-3 ligand (PeproTech, Cranbury, NJ) and 15 ng/mL interleukin-3 (R&D Systems, MN)) at a density of 7.5 × 10^4^/cm^2^ of surface area for 1–2 days on confluent MSCs in a humidified 5% CO_2_ atmosphere at 37 °C. Afterward, they were collected and cultured on sub-confluent MSCs (see below).

#### KG-1a cells and co-culture

The CD34^+^ acute myelogenous leukemia cell line KG-1a (DSMZ no. ACC 421, Leibniz Institute DSMZ–German Collection of Microorganisms and Cell Cultures) was cultured in medium (RPMI 1640, #21875034, Gibco) containing 10% FCS at 37 °C in a humidified 5% CO_2_ atmosphere. For CXCR4 inhibition and chemokine treatments they were incubated with either AMD3100 (1 or 10 µM), SDF-1 (100 ng/mL) or a combination of both for 8 h prior to video or confocal laser scanning microscopy (CLSM) (see below). For the experiment with the sub-confluent MSCs, KG-1a cells (2 × 10^4^) or CD34^+^ HSPCs (3 × 10^4^) were seeded and cultured for a given time (e.g., 4 or 12 h) as indicated in the corresponding legends. MSCs were pre-cultured for 24 h before the addition of hematopoietic cells. Cells were treated for 4 h with AMD3100 and/or SDF-1 as described above before imaging by phase contrast microscopy. The MSC media or serum-free HSPC media were used for the co-culture of KG-1a cells and CD34^+^ HSPCs, respectively.

### Plasmids and cell transfection

The expression plasmids, GFP-rab7 WT and pcDNA3-CXCL12-sfGFP were acquired from Addgene (#14436 and #98961, respectively). MSCs (5 × 10^5^) were transiently transfected by electroporation with 2 µg of plasmid DNA using an Amaxa Nucleofector 2B with Human Mesenchymal Stem Cell Nucleofector™ Kit (#VVPE-1001, Lonza Biosciences) according to manufacturer protocols. Cells were incubated at 37 °C for 5 min post-transfection and seeded on 6-well plates to be cultured for 24 h prior to be processed for downstream experiments. Under these conditions, approximately 3–5% of the transfected cells ectopically expressed the transgene.

### Fluorescence-labeling and confocal laser scanning microscopy

MSCs growing on fibronectin-coated glass coverslips (5 µg/cm^2^) (or other substrata as indicated) were labeled for various protein markers using specific primary antibodies (Additional file 1: Table S1) and appropriate fluorochrome-coupled secondary antibodies (Additional file 1: Table S2) and/or probed with fluorophore-coupled wheat germ agglutinin (WGA). Briefly, for all antigens except SDF-1, cells were washed with Ca^+^/Mg^+^ PBS (PBS containing 1 mM CaCl_2_ and 0.5 mM MgCl_2_) before fixation with 4% paraformaldehyde (PFA) for 20 min at room temperature, and then quenched with 50 mM NH_4_Cl for 10 min. Cells were either permeabilized with 0.2% saponin (AppliChem GmbH) in blocking buffer (PBS containing 0.2% gelatin) for 30 min [38] or incubated in blocking buffer without saponin (non-permeabilized conditions). For the SDF-1 immunostaining, the cells were fixed with ice-cold methanol at − 20 °C for 20 min, followed by 2 washes with ice-cold PBS. [Note that the SDF-1 immunolabeling was not successful with PFA-fixed cells]. Afterwards, for both protocols, samples were incubated with appropriate primary antibodies diluted in blocking buffer for 30 min, washed thrice with blocking buffer and then incubated with appropriate fluorochrome-conjugated secondary antibody (1:400) diluted in blocking buffer for 30 min at room temperature. Samples were subsequently incubated with CF®488A or CF®640R conjugated WGA (1:400, #29022 and #29026, respectively, Biotium, Fremont, CA) diluted in Hank’s balanced salt solution (HBSS, #14025100, Gibco) for 30 min. For labeling of actin cytoskeleton, cells were stained with fluorophore-conjugated phalloidin (1:400) diluted in PBS. KG-1a cells were immunolabeled for CXCR4 after incubation with or without SDF-1 or AMD3100 as described above. Samples were observed with a Zeiss LSM 700 laser scanning confocal microscope (Carl Zeiss, Jena, Germany). Various Zeiss objectives were used (40x/1.3 oil and 63x/1.4 oil). The images acquired under same setting for all cell lines were processed with Fiji and figures were prepared with Adobe Illustrator (Adobe Inc, USA).

### Scanning electron microscopy

MSCs growing on fibronectin-coated coverslips were fixed in 2% glutaraldehyde for 1 h at room temperature and then overnight at 4 °C. Following 2-h post-fixation in 1% osmium tetroxide at 4 °C, cells were subjected to dehydration in an acetone gradient (25–100%) and critical-point dried in a CO_2_ system (Automated Critical Point Dryer, Leica Microsystems, EM CPD 300, Wetzlar, Germany). Samples were then sputter-coated with gold (Sputter Coating Device SCD 050, BAL-TEC GmbH, Witten, Germany) and examined at 5-kV accelerating voltage in field emission scanning electron microscope (Jeol, JSM-7500 F, Japan).

### Time-lapse video microscopy

Cells were seeded on fibronectin-coated glass-bottom dishes and cultured for 24 h (MSCs) or 8 h (KG-1a cells) before imaging. For co-culture imaging, KG-1a cells were added on MSCs that were seeded 24 h prior. Growth medium was replaced with fresh medium with or without desired treatments before live-cell imaging. Imaging was performed with a widefield fluorescent microscope (Zeiss Axiovert 200 M, 20x/0.8 Ph2 objective, Jena, Germany). The microscope was equipped with an incubation chamber allowing imaging at 37 °C under 5% CO_2_ atmosphere. Images were taken at 15-min intervals over a period of 4, 8 or 12-h. Cell tracking was evaluated using TrackMate through Fiji [39, 40].

### Total internal reflection fluorescence (TIRF) microscopy

MSCs growing on fibronectin-coated glass-bottom dishes were stained for actin-cytoskeleton with 500 nM SiR-Actin dye (#SC001, Spirochrome, Switzerland) for 4 h before imaging. Cells were observed in a Leica DMI6000 microscope equipped with a 100x/1.46 oil immersion objective under cell culture conditions. Images were obtained at 1-min intervals using a penetration depth of 250 nm using a TIRF module.

### Statistical analysis

Statistical analyses were performed in R software (Version 4.2.0, R Foundation, Vienna, Austria). For comparison of percentage of cells harboring retraction fibers with or without migrasomes, a chi-square test with Yates’ correction were used. A Mann-Whitney U test was used for comparing population medians of cells with migrasomes and KG-1a migration distances. Quantification of cell density was performed by (1) outlining regions containing MSCs, migrasome network, and fibronectin-coated free areas followed by calculating their surface area in mm^2^; (2) counting the number of hematopoietic cells associated with these regions and then calculating their density per mm^2^; and (3) determining fold changes by dividing the density of hematopoietic cells on MSCs or migrasome networks by their density on the free surface. A two-tailed *T* test was then used to compare the fold changes in KG-1a cell or CD34^+^ HSPC density in MSC co-cultures. All data are shown as the mean ± standard deviation (S.D.) of at least three independent experiments.

## Results

### MSCs produce a migrasome network by two distinct cellular mechanisms

**Fig. 1:**
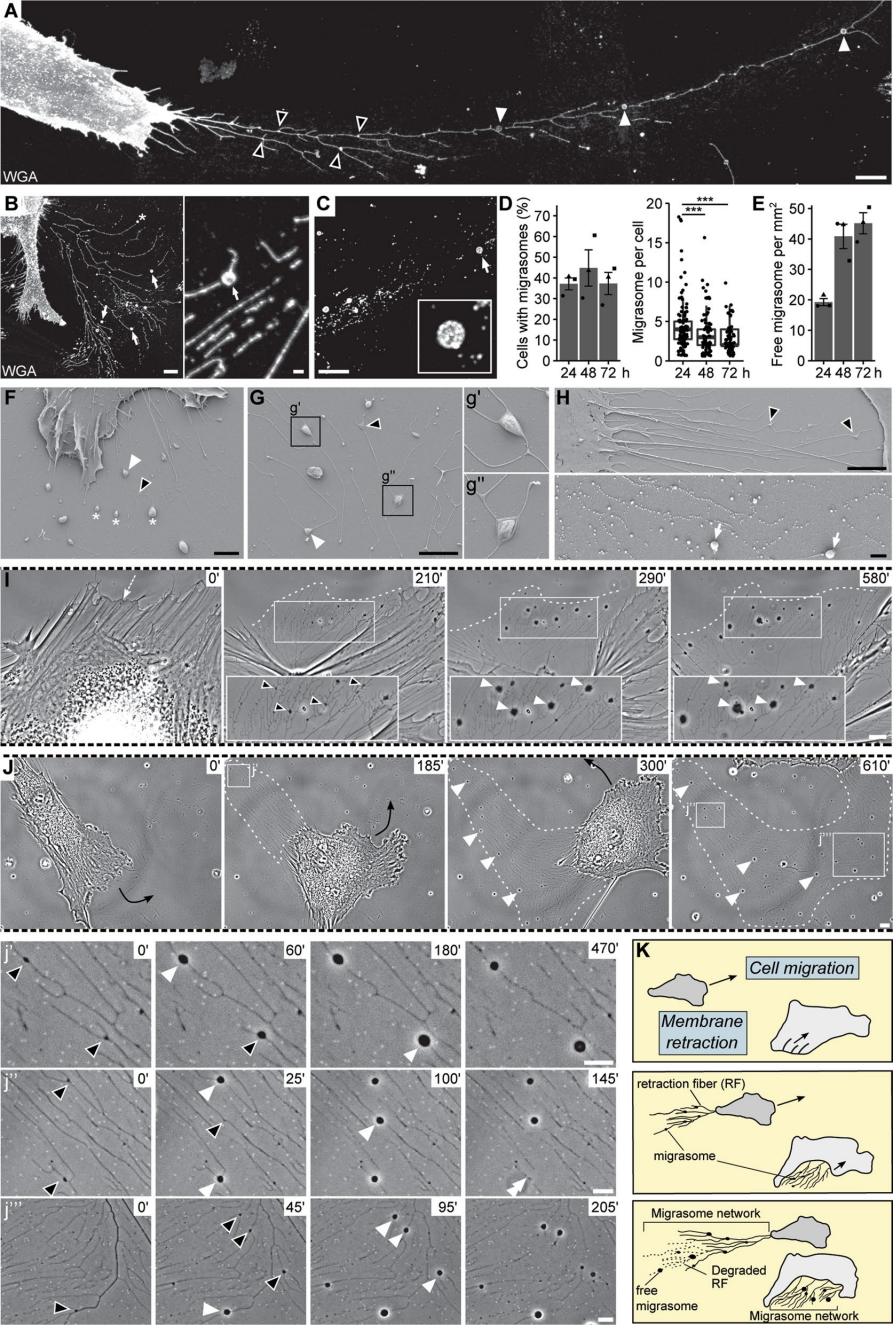
Primary human mesenchymal stromal cells produce migrasome networks. **A**–**J** Primary human MSCs were cultured on fibronectin-coated glass coverslips for 24–72 h before being processed for CLSM (**A**–**E**), SEM (**F**–**H**), or live-cell phase-contrast video microscopy (**I**, **J**). After 24 h in culture, PFA-fixed cells were stained with fluorophore-conjugated WGA (**A**–**C**). Early-stage and fully formed migrasomes that develop along the retraction fibers left behind a migrating MSC are indicated by black and white arrowheads, respectively. Migrasomes are also found at the tips of retraction fibers (**B**, asterisk). Over time, the retraction fibers breakdown, leaving the migrasomes free (**B**, **C**, arrow and inset). The number of cells with migrasomes and the number of migrasomes per cell (**D**) were quantified after 24, 48, and 72 h of culture. Cell-free, detached migrasomes per mm^2^ were quantified over time in cultured cells (**E**). The mean and S.D. of all data are shown in bar plots and symbols show the values of a given experiment (**D**, left, **E**), while box-and-whisker plots show data from 25 to 75th percentiles and 95% within the whiskers. Horizontal line represents the median, where each dot represents a cell (**D**, right) (> 150 cells per replicate, *n* = 3). 48-h cultured MSCs were processed for SEM (**F**–**H**). Note that migrasomes often develop on the branching points of the retraction fibers or at their tips (**F**, **G**, inset g′, g”, **H**, upper panel) and are released upon degradation of the fiber network (**H**, lower panel). The formation of migrasomes is revealed by time-lapse video imaging of MSCs (**I**, **J**). After 24 h in culture, the dynamics of MSCs and the growth of migrasomes were recorded for a period of 12 h. Elapsed time in minutes is shown on the top-right corner. Dashed white and black arrows indicate the direction of MSC membrane retraction or the cell migration, respectively, while dashed outline shows the extent of the migrasome network. The migrasome maturation is highlighted over time in panels **J** (insets) and **J** (insets j′, j″, j‴) and their release is indicated (double arrowhead). The images presented in **I** and **J** are excerpted from the Additional file 2: Video S1 and Additional file 3: Video S2, respectively. **K** Migrasome biogenesis by MSCs is triggered by two distinct cellular mechanisms: cell migration and membrane retraction. In both cases, migrasomes grow on the retraction fibers and are released upon degradation of the fiber network. Data were compared using either Mann–Whitney U test (**D**, **E**). ***p < 0.001. Scale bars, 10 μm (**A**–**C**, **I**, **J**); 5 μm (**H**, upper panel); 1 μm (**F**, **G**, **H**, lower panel, j′–j‴)

 In our study of the interaction between cancer cells and stromal cells, such as those residing in the bone marrow microenvironment, we found that plastic-adherent primary human MSCs growing on fibronectin (5 µg/cm^2^)-coated glass coverslips for 24 h produce a new type of cellular bulges, called migrasomes, along the retraction fiber network (Fig. 1A). These structures, observed by fluorochrome-conjugated WGA staining that labeled glycoconjugates at the cellular membrane, appeared either at the branching points or along the retraction fibers, as well as at the terminal end (Fig. 1A, B, asterisks). Structurally, they are small and large in their appearance (Fig. 1A, black and white arrowheads, respectively), suggesting that they are dynamic structures (see below). Cell-free and detached migrasomes were also observed notably when retraction fibers are degraded which occurs over time (Fig. 1C). Quantification revealed that approximately 25–40% of MSCs harbored migrasomes, regardless of the time in culture, i.e. from 24 to 72 h (Fig. 1D, left panel). Nevertheless, the number of migrasomes per cell decreased during this period, while the amount of cell-free, detached migrasomes increased (Fig. 1D, right panel, E). Retraction fiber-associated and cell-free migrasomes, small and large, can be observed at high resolution using scanning electron microscopy (SEM) (Fig. 1F–H and corresponding insets). Time-lapse video-microscopy revealed the dynamic maturation of migrasomes, i.e., from early-stage (small, triangular shape) to fully formed (large, rounded shape) migrasomes, along the retraction fibers (Fig. 1I, J and corresponding insets, Additional file 2: Video S1, Additional file 3: Video S2). Two main mechanisms underlying the migrasome formation are highlighted by these live-cell images. They develop on retraction fibers that have emerged from the membrane retraction of a non-migrating cell (Fig. 1I) or those left behind a migrating MSC (Fig. 1J). In both cases, migrasomes along the retraction fibers can create a “migrasome network” as a track behind migrating cells or surrounding a non-migrating cell (Fig. 1K, for detail see figure legend). Under these conditions, migrasomes were observed in MSCs derived from 4 independent donors (data not shown).

**Fig. 2:**
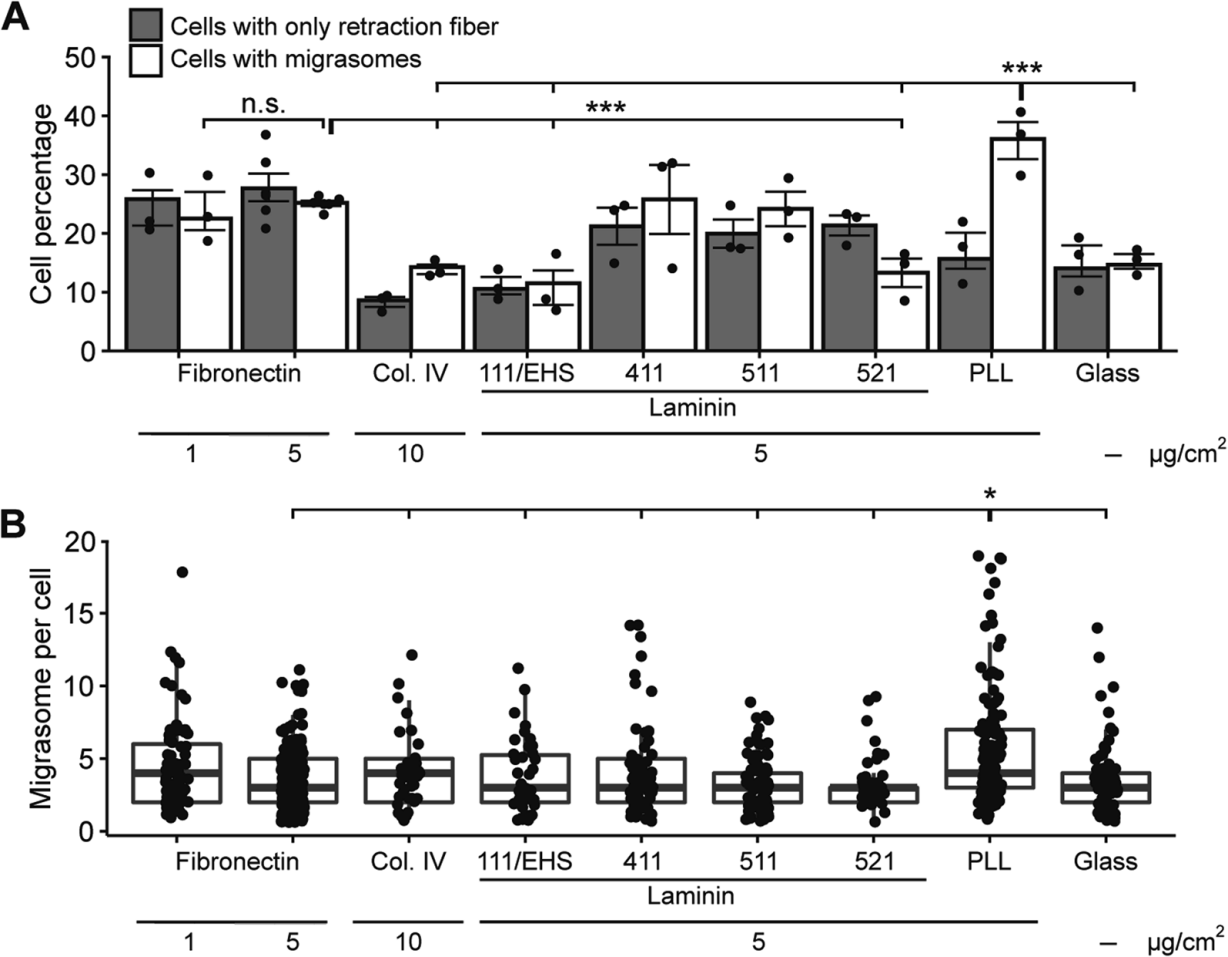
Impact of matrix substrates on migrasome formation. **A**, **B** Primary human MSCs were cultured on different substrates at different concentrations as indicated or on uncoated glass surfaces for 24 h before being processed for CLSM. PFA-fixed cells were stained with fluorophore-conjugated WGA. Cells producing only retraction fibers and those with migrasomes were quantified (**A**, > 150 cells per replicate, *n* ≥ 3). The number of migrasomes per cell were quantified (**B**, > 50 cells per replicate, *n* ≥ 3). The mean and S.D. are shown in bar plots and symbols show the values of a given experiment (**A**), while boxes in plots represent data from 25–75th percentiles and middle line showing the median where each dot represents a cell (**B**). Data were compared using either Chi-Square test with Yates’ correction (**A**) or Mann–Whitney U test (**B**). N.s., not significant, *p < 0.05, ***p < 0.001

Then, we evaluated different matrix substrates for the potential of MSCs to produce migrasomes. To that end, MSCs were cultured for 24 h on glass-coverslips coated with fibronectin (1 or 5 µg/cm^2^), collagen IV (10 µg/cm^2^), laminin (5 µg/cm^2^), or PLL (5 µg/cm^2^) prior their fixation and labeling (Fig. 2). In the case of laminin, we used four distinct ones harboring different α and β chains; laminin-111/EHS, laminin-411, laminin-511, laminin-521 [41–43]. About 50% of MSCs harbored retraction fibers with or without migrasomes when cultured on fibronectin, irrespective of its concentration, and on laminin-411 and -511 (Fig. 2A). This number drops significantly to 30% when uncoated surfaces such as glass is used. Similarly, numbers of MSCs with retraction fibers containing migrasomes or not were reduced on collagen IV, laminin-111/EHS and -521. In the case of collagen IV, and to a lesser extent on laminin-411/-511, we observed more retraction fibers containing migrasomes than those without, which contrasts with other substrates or uncoated surfaces where approximately 50% of retraction fibers are found with migrasomes. Interestingly, when PLL is used, the presence of migrasomes along the retraction fibers is greatly increased (Fig. 2A). In addition, quantification of the number of migrasomes per cell showed that their presence is significantly increased on PLL compared to all other substrates (Fig. 2B), suggesting that strong attachment is a prerequisite for their formation. Of note, it appears that most migrasomes emerged either from retraction fibers produced by membrane retraction of non-migrating MSCs as in the case of those growing on PLL, or from retraction fibers left behind migrating MSCs as in the case of laminin-411 (data not shown).

### MSC-associated migrasomes contain F-actin and tubulin

**Fig. 3:**
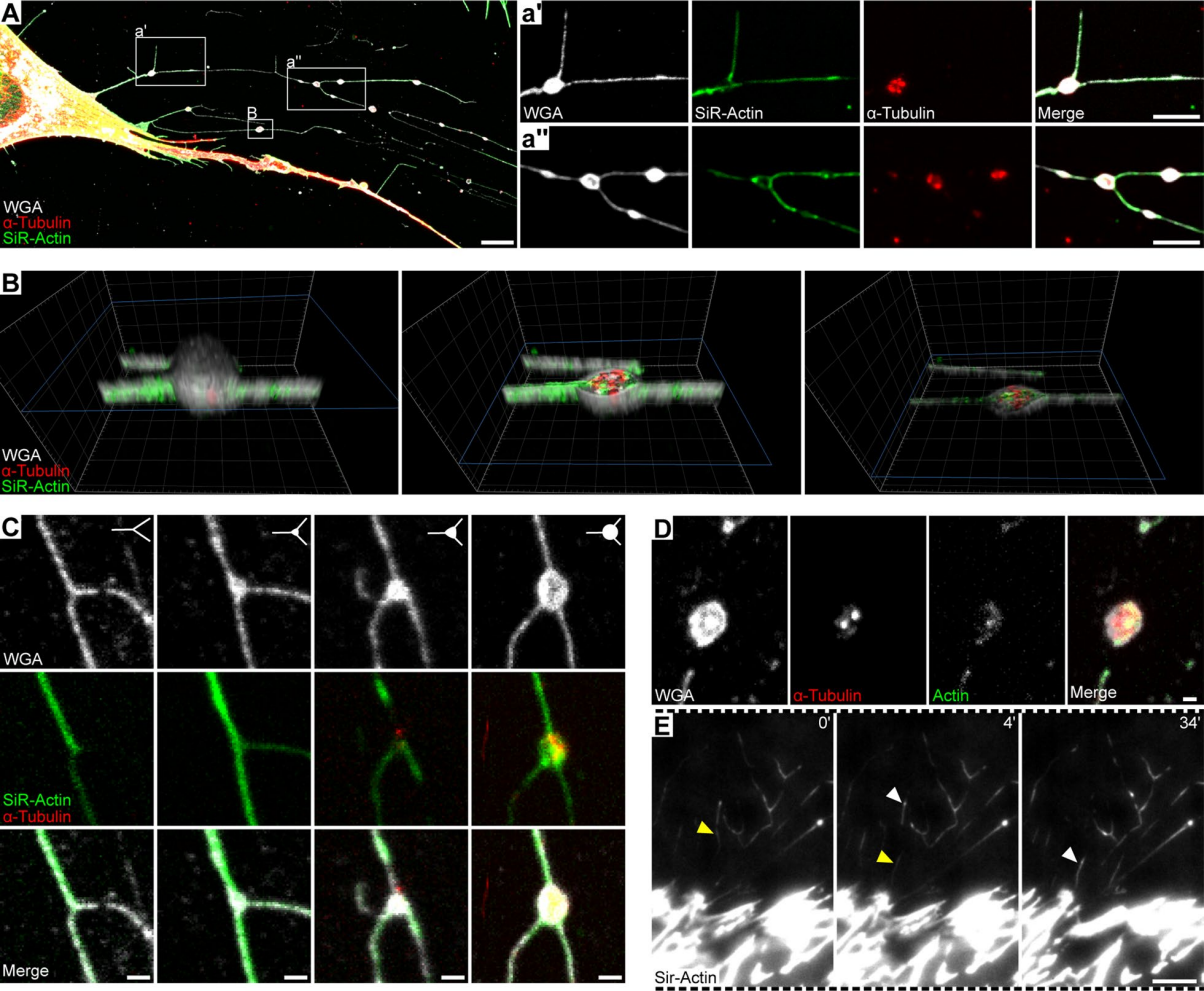
F-actin is associated with both retraction fibers and migrasomes and tubulin is restricted to migrasomes. **A**–**E** Primary human MSCs were cultured on fibronectin-coated glass coverslips for 24 h before being processed for CLSM (**A**–**D**) and total internal reflection fluorescence (TIRF) microscopy (**E**). PFA-fixed cells were saponin-permeabilized prior immunolabeling with anti-α-tubulin antibodies and staining by SiR-Actin (**A**–**C**, **E**) or phalloidin (**D**) and fluorophore-conjugated WGA, which label F-actin and cellular membrane, respectively. The distribution of F-actin and tubulin in retraction fibers and migrasomes shown in **A** is highlighted in the insets (a′, a″). A 3D render of a single migrasome highlights the presence of actin and tubulin therein (**B**). The distribution of cytoskeleton constituents in early-stage and fully formed migrasomes (**C**, from left to right panels, respectively, see also Additional file 1: Fig. S1) and in cell-free, detached migrasome (**D**) is shown. The movement of SiR-Actin-stained actin filaments in retraction fibers of living cells is recorded by TIRF video microscopy at a layer height of 250 nm (**E**). White and yellow arrowheads point the movement of actin filaments within the time frame presented. Elapsed time in minutes is shown on the top-right corner. The images presented in **B** and **E** are excerpted from the Additional file 4: Video S3 and Additional file 5: Video S4, respectively. Scale bars, 10 μm (**A**, **E**); 5 μm (a′, a″); 1 μm (**C**, **D**)

To obtain information on migrasome composition, including cytoskeletal organization and dynamics, MSCs were stained with SiR-Actin, a cell permeable fluorogenic agent that specifically labels F-actin, and were immunostained for α-tubulin. Cells were counterstained with WGA to highlight the overall structure. CLSM analysis revealed the presence of actin in the retraction fibers and migrasomes associated with them, whereas α-tubulin was restricted to migrasomes (Fig. 3A, inset a′ and a″). Note that the distribution of F-actin in the retraction fibers is not uniform, suggesting its dynamic localization. The 3D rendering of a migrasome showed the F-actin and α-tubulin in it (Fig. 3B, Additional file 4: Video S3). Analysis of immature and mature migrasomes revealed that α-tubulin appeared in the later stages of migrasome formation (Fig. 3C, Additional file 1: Fig. S1). The F-actin in both retraction fibers and migrasomes of PFA-fixed MSCs is also detected with fluorochrome-conjugated phalloidin instead of SiR-Actin (Fig. 3D). Due to its heterogenous distribution along the retraction fibers, the dynamics of F-actin was observed in live cells by TIRF microscopy, highlighting the actin transport along the retraction fibers and migrasomes (Fig. 3E, Additional file 5: Video S4).

Next, we investigated the presence of integrins in migrasomes. Interestingly, both members of α5β1 dimer (CD49e/CD29), also known as the fibronectin receptor, were found in migrasomes and the associated retraction fibers (Additional file 1: Fig. S2A), as reported for cancer cells [44]. These data are consistent with a higher number of cells harboring a migrasome network when growing on fibronectin (see above). Integrins α2β1 (CD49b/CD29) that act as a receptor for collagen were detected therein although weaker in the case of α2 (Additional file 1: Fig. S2A). In contrast, integrin α2b (CD41a), α4 (CD49d), α6 (CD49f), αE (CD103), αM (CD11b), αX (CD11c), β2 (CD18) and β4 (CD104) were absent (or extremely weak as in the case of β3 (CD61)) in the migrasome network (Additional file 1: Fig. S2A), which is in agreement with our general cell surface proteome analysis of MSCs [45]. Melanoma cell adhesion molecule (MCAM, CD146), which may act as a laminin receptor [46, 47], is also expressed in retraction fibers and migrasomes (Additional file 1: Fig. S2A). Although the same data were obtained when MSCs were grown on PLL instead of fibronectin (Additional file 1: Fig. S2B, data not shown), integrin α6 is very weakly upregulated when laminin-411 and -511 are used as substrata, whereas integrin α5 partially reduced (Additional file 1: Fig. S2C, D).

### MSC-associated migrasomes contain late endosomes/multivesicular bodies, but no mitochondria

**Fig. 4:**
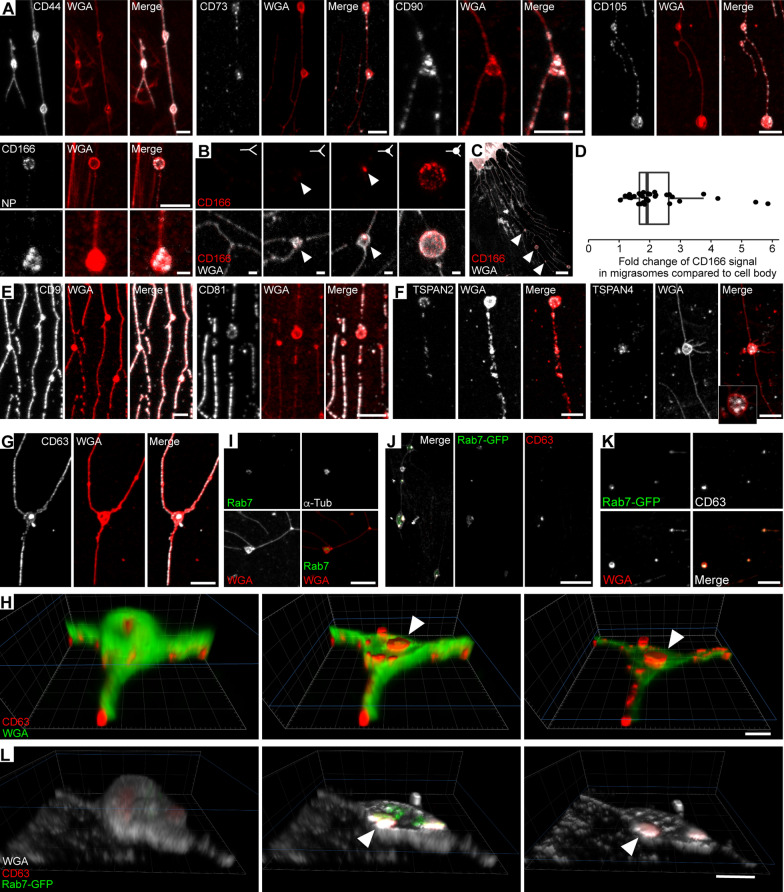
MSC-derived migrasomes exhibit a distinctive cell surface CD marker profile and carry intracellular vesicles. **A**–**I** Primary human MSCs were cultured on fibronectin-coated glass coverslips for 24 h before being processed for CLSM. PFA-fixed cells were permeabilized with saponin or non-permeabilized (NP) before immunolabeling for a given CD marker (**A**–**C**, **E, G**), specific tetraspanin proteins (TSPAN 2 and 4, **F**), Rab7 or α-tubulin (**I**) as indicated followed with the appropriate fluorophore-conjugated secondary antibody and membrane staining with fluorophore-conjugated WGA. CD166 immunolabeling shows its presence in mid- and fully formed migrasomes (**B**, **C**, arrowheads), whereas it is absent or very weakly expressed in early stage migrasomes (**B**). Quantification of CD166 signal in migrasomes and its comparison to cellular protein values show elevated levels of CD166 protein in migrasomes (**D**). The box-and-whisker plot shows data from 25 to 75th percentiles and 95% within the whiskers. Horizontal line represents the median, while each dot denotes a single migrasome. 3D render of a single migrasome presented in **G** highlights the presence of an intracellular pool of CD63 (**H**, arrowhead). **J**–**L** Transiently transfected Rab7-GFP MSCs were immunolabeled for CD63 and stained with fluorophore-conjugated WGA (white in **J**). A 3D render of the migrasome presented in **J** highlights the presence of cytoplasmic CD63 and late endosomal marker Rab7 (**L**, arrowhead). Cell-free, detached migrasomes are displayed (**K**). The images presented in **H** and **L** are excerpted from the Additional file 6: Video S5 and Additional file 7: Video S6, respectively. Scale bars, 5 μm (**A**–**C**, **E**–**G**, **I**–**K**); 2 μm (**L**); 1 μm (**A**, CD166, lower panel, **H**)

Since migrasomes and retraction fibers create an extensive network either behind migrating cells or around non-migrating cells, it is of interest to determine whether certain MSC cell surface markers are expressed in this cellular meshwork. We investigated the presence of CD44, CD73 (5′-nucleotidase), CD90 (THY-1), CD105 (endoglin) and CD166 by immunolabeling [4, 45, 48]. Five mentioned markers were detected in MSCs cultured on fibronectin-coated glass coverslips. All of them, except CD166, were found in both retraction fibers and migrasomes (Fig. 4A). CD44 appeared homogeneously along the retraction fibers, whereas CD90 and CD105 showed punctate staining (Fig. 4A). CD73, which have been proposed as a prospective marker to isolate MSCs [49], displayed a sparse, dotty presence along the retraction fibers (Fig. 4A). CD166, a transmembrane adhesion protein involved in homophilic and heterotypic interactions [50], is known to regulate the hematopoietic stem cell niche and angiogenesis [51, 52]. This protein is selectively concentrated in migrasomes being barely detectable in retraction fibers (Fig. 4A). It appeared in migrasomes at early stage of their formation, i.e. before the appearance of tubulin therein (Fig. 4B). Interestingly, quantification of CD166 immunolabeling on the surface of mature migrasomes revealed its selective enrichment at this location relative to the cell surface (Fig. 4C, D). Tetraspanin CD9, an interaction partner of CD166 that regulates its adhesive properties [53], was also detected in migrasomes in addition to retraction fibers (Fig. 4E). Four other tetraspanin proteins (CD63, CD81, TSPAN2, and TSPAN4) were detected in these networks (Fig. 4E, F). TSPAN4, like CD166, was specific for migrasomes (Fig. 4F). CD63 was found on the surface of migrasomes as well as within them (Fig. 4G). CD63 is a marker of small intralumenal vesicles found in LE/MVB, which are released as exosomes after the fusion of LE/MVB with the plasma membrane [54]. The 3D rendering of a migrasome highlights the presence of CD63 in the internal vesicles (Fig. 4H, Additional file 6: Video S5). To confirm the presence of LE/MVB in migrasomes, we either labeled MSCs with an antibody directed against the small GTPase Rab7 or transiently transfected them using a plasmid encoding the Rab7-GFP fusion protein. Both approaches revealed the presence of Rab7 in migrasomes (Fig. 4I, J). Co-labeling of CD63 with Rab7-GFP revealed their presence in migrasomes (Fig. 4J, K, Additional file 1: Fig. S3A) and the 3D rendering shows their sub-migrasomal localization (Fig. 4L, Additional file 7: Video S6). Interestingly, their presence in migrasomes persists when the latter structures detach from the cells after the degradation of the retraction fibers (Fig. 4K), suggesting that some cellular components of MSCs may have a role when they are not in direct contact with the cell itself.

Recently, mitochondria have been shown to localize to migrasomes under oxidative stress in mouse fibroblasts and neutrophils [55]. Here, mitochondria were absent from MSC-associated migrasomes under native and oxidative stress conditions, regardless of whether the cells were grown on fibronectin or PLL substrates (Additional file 1: Fig. S3B).

### MSC-associated migrasomes contain signaling molecules

**Fig. 5:**
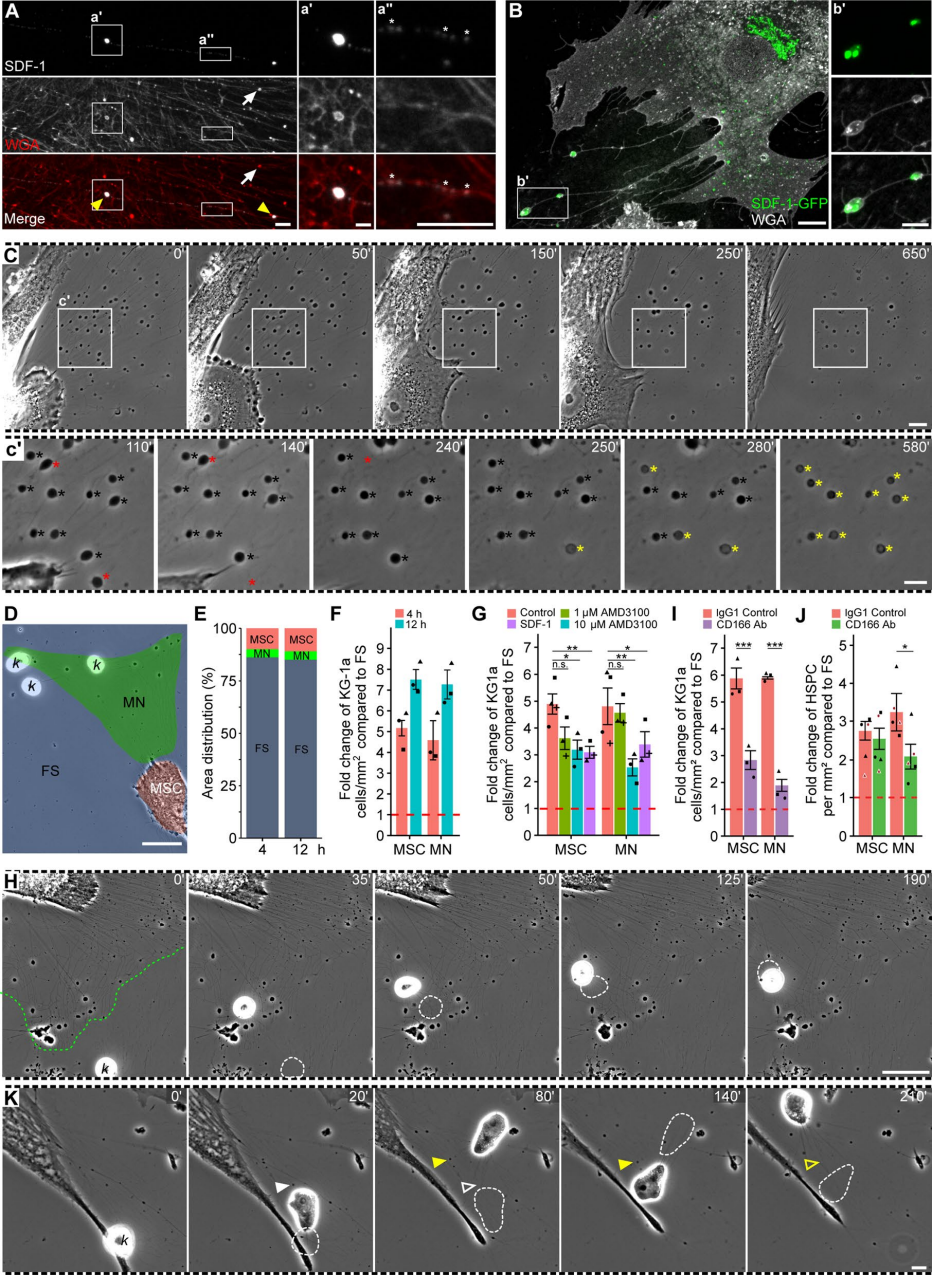
MSC-derived migrasomes can act as a chemoattractant organelle. **A**–**K** Primary human MSCs were cultured on fibronectin-coated glass coverslips under sub-confluent state for 24 h prior to, either, imaging (**A**–**C**), or addition of KG-1a (*k*) cells (**D**–**I**, **K**) or primary CD34^+^ HSPCs (**J**) for 4 (**E**–**G**, **I**, **J**) or 12 (**D**–**F**, **H**, **K**) h co-culture as indicated. Afterward, they were processed for CLSM (**A**, **B**) or live-cell microscopy (**C**–**K**). CD34^+^ HSPCs were used 24 (red) or 48 (black) h after their immunoisolation (**J**). SDF-1 was detected by immunolabeling (**A**) or upon transient transfection with SDF-1-GFP (**B**), followed by staining with fluorophore-conjugated WGA. SDF-1 can be observed in migrasomes (yellow arrowhead, a′, b′) or retraction fibers (a″, asterisk). Note that not all migrasomes contained SDF-1 (white arrows). Time-lapse video of an MSC-associated migrasome network shows the migrasomes detaching from substrate (**C**, c′, red asterisks) or releasing their contents into medium as suggested by phase-shift (c′, black → yellow asterisks). The boxed regions in **C** indicate the areas enlarged in the insets. The elapsed time in minutes is shown on the top right corner. All frames are excerpted from Additional file 9: Video S8. The areas covered by MSCs (MSC), the migrasome network (MN), and free surface (FS) are shown (**D**, **E**), while the distribution of KG-1a cells or CD34^+^ HSPCs on these regions was quantified after 4 (**E**, **F**, **J**) or 12 (**D**–**F**) h of co-culture with MSCs. Data are presented as the fold change of the density of KG-1a cells or CD34^+^ HSPCs per mm^2^ in regard to FS; dashed red line indicates same density with FS, thereby no preferential localization (**F**–**J**). KG-1a cells were co-cultured with MSCs in the presence of either 1 or 10 µM AMD3100 or 100 ng/mL recombinant SDF-1 for 4 h and their distribution were quantified as in panel F (**G**). Alternatively, MSCs were pre-treated with anti-CD166 antibody, or IgG control, for 2 h prior to addition of KG-1a (**I**) cells or CD34^+^ HSPCs (**J**). Error bars shows the S.D., while symbols show the individual experiments (**F**, **G**, **I**) or donors (**J**). More than 200 cells were analyzed for each experiment (*n* = 3). Mean of experiments were compared against control using unpaired *T* test (**G**, **I**) or paired *T* test (**J**, note that only 48-h values were used for statistical analysis). Migrating KG-1a cells can either move towards a migrasome network and be captured by retraction fibers (**H**) or uptake the migrasomes during their movement (**K**, arrowheads). Green and white dashed outlines show the position of migrasome network (**H**) and the cell in the previous frame (**H**, **K**), respectively. White and yellow arrowheads show the migrasomes that are taken by KG-1a cell (**K**). All frames are excerpted from Additional file 10: Video S9 and Additional file 11: Video S10, respectively. N.s., not significant, *p < 0.05, **p < 0.01, ***p < 0.001. Scale bars, 10 μm (**C**, **D**, **H**, **K**); 5 μm (**A**, **B**, a″, c′); 1 μm (a′, b′)

To better understand the role of MSC-associated migrasomes, particularly in the context of intercellular signaling, we investigated whether the chemoattractant SDF-1 was present. Again, we used two distinct approaches based on immunostaining and expression of a fusion protein (Fig. 5A, B). SDF-1 immunoreactivity was detected in migrasomes (Fig. 5A, inset a′) and in a punctate pattern along retraction fibers (Fig. 5A, inset a″), suggesting that SDF-1 is actively transported to retraction fiber-associated migrasomes. Similarly, ectopic expression of SDF-1-GFP revealed its incorporation into migrasomes attached to retraction fibers (Fig. 5B, inset b′). Cell-free, detached migrasomes also contained SDF-1-GFP (Additional file 1: Fig. S4, Additional file 8: Video S7). Of note, only a fraction of migrasomes contains SDF-1 (i.e., about 13% of all migrasomes; 60 migrasomes, *n* = 10 cells) as revealed by either immunostaining or ectopic expression suggesting that its accumulation therein is regulated. As a chemoattractant, SDF-1 is secreted by MSCs [14] and, in the context of the bone marrow stem cell niche, it plays a role in the process of HSPC homing. Observations by phase-contrast microscopy showed sudden changes in the phase-shift of migrasomes (Fig. 5C, inset c′, black → yellow asterisk, Additional file 9: Video S8), which can be explained by loss of their membrane integrity, suggesting release of their cargo. The latter might explain the small proportion of migrasomes containing SDF-1. In addition to the release of their contents, migrasomes were also released into the conditioned medium (Fig. 5C, inset c′, red asterisk).

### MSC-associated migrasomes attract leukemic KG-1a cells and primary CD34^**+**^**HSPCs**

To assess the impact of the MSC-associated migrasome network on cells of hematopoietic origin, we co-cultured MSCs growing on fibronectin-coated glass coverslips with leukemic KG-1a cells for 4 and 12 h and then assessed the amount of cancer cells associated with the cells and/or migrasome network. First, quantification of the surface areas covered by the cells or migrasomes revealed that they occupied less than 25% of the total surface area, which was consistent between the observed time-points (Fig. 5D, E). Interestingly, the distribution of leukemic cells revealed a strong preference for MSCs and migrasome network over the cell-free areas as time progressed (Fig. 5F). Thus, KG-1a cells preferentially attached to resident MSCs and their migrasomes rather than to free surfaces coated with fibronectin as an extracellular matrix (ECM) component, suggesting that the release of factors such as the chemoattractant SDF-1 attracted them and/or adhesive protein retained them. Further, KG-1a cells were observed to express CXCR4 (Additional file 1: Fig. S5A), a key protein that tethers hematopoietic cells to the bone marrow cells [56], which further supports this hypothesis. To evaluate that, we added AMD3100, a specific inhibitor of CXCR4, to the co-cultured cells. The amounts of leukemic cells interacting with MSCs or the migrasome network were significantly reduced in the presence of 10 µM AMD3100 (Fig. 5G), indicating that the CXCR4–SDF-1 axis is involved in the selective localization of KG-1a cells. At 1 µM AMD3100, a minor effect was observed only with MSCs, although not statistically significant (*p* = 0.07), suggesting, albeit indirectly, that the migrasome network secreted a greater amount of SDF-1. Tracking of KG-1a cells by time-lapse video microscopy for a period of 8 h in the absence or presence of AMD3100 showed that the drug did not affect their migration at any concentration (Additional file 1: Fig. S5B, C). The implication of CXCR4–SDF-1 axis was further investigated by adding recombinant SDF-1 (100 ng/mL) to the co-culture media (Fig. 5G). This concentration of SDF-1 was shown to induce the polarization of CXCR4 at the plasma membrane of KG-1a cells as well as increase their migration (Additional file 1: Fig. S5A, B, respectively), while these effects of SDF-1 were partly nullified upon addition of AMD3100 indicating the increased migration was directly linked to CXCR4–SDF-1 axis. Addition of recombinant SDF-1 significantly decreased the amount of leukemic cells associated with both MSCs and migrasome network (Fig. 5G), suggesting that the SDF-1 gradient created by MSCs and migrasomes is attenuated. Indirectly, these experiments suggest that the accumulation of cancer cells on MSCs or migrasomes is not a random event. The directed migration of a KG-1a cell to a migrasome and its retention therein is shown by time-lapse video-microscopy (Fig. 5H, Additional file 10: Video S9). Note that under these conditions, neither AMD3100 nor recombinant SDF-1 impact the MSC-associated migrasomes (data not shown).

Next, we investigated the role of the cell adhesion molecule, CD166, in the selective retention of KG-1a cells on either MSCs or migrasome networks. To that end, MSCs were preincubated with either anti-CD166 antibody (5 µg/mL) or the mouse IgG control antibody for 2 h, followed by removal of antibodies and introduction of KG-1a cells for 4 h. Interestingly, a significant reduction of KG-1a cells associated with both MSCs and migrasome networks were observed (Fig. 5I). Of note, the effect of the anti-CD166 antibody appears to be more profound on the migrasome network than on MSCs, suggesting that the selective concentration of CD166 on migrasomes plays a role in the retention of leukemic cells therein.

Apart from the leukemic cells, we evaluated whether the MSC-associated migrasomes impact the localization of primary CD34^+^ HSPCs. The latter were immunoisolated from mobilized peripheral blood using MACS technology and then cultured for 24 or 48 h before the experiments. Similar to KG-1a cells, they showed the same tendency to be attracted to MSCs and migrasome networks, regardless of their culture time after immunoisolation (Additional file 1: Fig. S6, controls). However, the difference in the density of CD34^+^ HSPCs associated MSCs or the migrasome network compared to free areas is more modest compared to leukemic cells, suggesting that their attraction and/or interaction with MSCs and migrasomes occurs to a lower extent. Nevertheless, the CXCR4–SDF-1 axis also appears to play a role in the enrichment of CD34^+^ HSPCs on MSCs and migrasome networks as demonstrated by the addition of CXCR4 inhibitor or recombinant SDF-1 (Additional file 1: Fig. S6).

Finally, the impact of CD166 was assessed as described above. In contrast to leukemic cells, the addition of the anti-CD166 antibody did not alter the preference of CD34^+^ HSPCs for MSCs themselves, but significantly reduced it for migrasome networks (Fig. 5J). The strong expression of CD166 on migrasomes (see above) and/or the presence or absence of CD166 interacting partner(s) therein or on hematopoietic cells might explain such differential impact between MSCs and their migrasome networks and between cancer cells and CD34^+^ HSPCs.

### MSC-associated migrasomes are selectively taken up by migrating leukemic cells but not by CD34^**+**^**HSPCs**

By investigating whether cells of hematopoietic origin are attracted in a CXCR4–SDF-1- dependent manner to the MSC-associated migrasome network, we noticed that migrasomes can be taken up by migrating leukemic cells. Time-lapse video-microscopy showed that migrating KG-1a cells can absorb both retraction fiber-attached or cell-free migrasomes during their movement (Fig. 5K, Additional file 1: Fig. S7, Additional file 11: Video S10, Additional file 12: Video S11). Under the same conditions, we did not observe that primary CD34^+^ HSPCs uptake migrasomes associated with MSCs. Instead, attracted CD34^+^ HSPCs continued their movement even after contacting migrasomes (Additional file 1: Fig. S8, Additional file 13: Video S12).

## Discussion

Intercellular communication is known to play roles during development and homeostasis. This is particularly true in the hematopoietic stem cell niches of the bone marrow, where many interactions between stromal cells, e.g., MSCs, and hematopoietic cells occur. Under pathological conditions, such as cancer, normal exchanges between healthy cells can be usurped by malignant cells, resulting in a transformation of the microenvironment that will promote cancer growth and metastasis. In addition to the classical signaling pathways involving soluble factors, other intercellular communication mechanisms, e.g., based on membrane structures, allow short- and long-distance communication between surrounding cells. For example, tunneling nanotubes and extracellular membrane vesicles (e.g., exosomes) have been described as allowing direct or indirect exchange of biomaterials between bone marrow-derived cells [19, 20, 57]. Here, we have shown that MSCs produce migrasomes, a unique structure growing on retraction fibers, which has been described to play a role in zebrafish development [31]. Together with other mechanisms of intercellular communication observed in hematopoietic stem cell niches, these observations add to the complexity of the cellular exchanges taking place thereon.

The biogenesis of MSC-associated migrasomes relies on the formation of retraction fibers that can be generated either by migrating or non-migrating cells. Structurally, they contain F-actin and α-tubulin. Two tetraspanin proteins (TSPAN2, and TSPAN4), previously described to be involved in the maturation of migrasomes [30] were found therein. Tetraspanins were proposed to form tetraspanin-enriched microdomains on retraction fiber membranes, which upon interaction with cytoskeleton and adhesion proteins, can assemble into macrodomains that can swell to form a migrasome due to local membrane rigidity differences [30]. The ECM components may influence such processes in addition to impact cell migration, and thus retraction fiber formation. Our data with different matrix substrata suggest a role of ECM as fibronectin, laminin-411 and -511 to favor the migrasome formation compared to collagen IV, laminin-111/EHS and -521. Fibronectin receptor (α5β1) may play a role when this ECM is used, while the slight increase in integrin α6 expression when MSCs are cultured on laminin-411 and -511, may explain the high number of retraction fibers with migrasomes on these substrata. In vivo, these laminins are highly relevant to the bone marrow stem cell niche [41–43]. Other integrins such as integrin α7 or CD146 may also be involved. The reduced amount of integrin α2, which participates with β1, as a receptor for collagen IV may explain, among other factors, the limited number of cells with migrasomes on this substrata. It remains to be determined whether these substrates can promote indirectly the growth of migrasomes themselves and/or their retention after retraction fiber degradation [44]. Indeed, the higher number of migrasomes per cell on PLL indicates that a stronger interaction between the retraction fibers and the ECM, could lead to a greater retention of migrasomes, rather than their release. These issues are important because migrasomes associated with retraction fibers and free migrasomes could create a cell-free microenvironment in the bone marrow niche, while retaining certain cellular components. The release of SDF-1 as a chemoattractant conferred a certain dynamic to these structures allowing hematopoietic cells to migrate, and then be trapped, in these networks. Thus, migrating MSCs can create a tract of migrasomes behind them that might guide other cells, e.g., within the bone marrow microenvironment. It remains to be evaluated whether MSC-associated migrasomes could impact the migration and/or retention of metastatic cancer cells in the bone marrow, and thus constitute a potential target for cancer therapy.

The concentration of the adhesion protein CD166 in migrasomes is of interest, as is CD9, which enhanced CD166 clustering and, therefore, stimulated intercellular adhesion [53]. As homophilic CD166 interactions are critical for HSPC engraftments and HSPC-niche interaction [52], these protein complexes could explain the adhesion of hematopoietic cells to migrasomes. The antibody blockage experiment is consistent with such a role. While the CD166 homotypic interactions can play a role in the adhesion of hematopoietic cells to migrasomes, the difference in the absorption of migrasomes observed between KG-1a cancer cells and CD34^+^ HSPCs might be a result, among others, of additional proteins that interact with CD166 [52]. One of these candidates might be CD6 [58–60], which is strongly expressed in KG-1 cells by comparison to CD34^+^ HSPCs [61, 62]. This could explain the difference or preference of cancer cells to MSCs and migrasome network over CD34^+^ HSPCs. Cell membrane dynamics between the cancer cells, i.e. KG-1a, and CD34^+^ HSPCs might also differ, as illustrated by the differential potential to form tunneling nanotubes [20]. In addition to MSC markers and integrins, it will be interesting to perform a comprehensive characterization of cell surface proteins associated with migrasomes and their cargoes including the presence of other signaling molecules and possibly microRNAs that could influence the fate of recipient cells that absorb them, notably the cancer cells. In this context, the presence of LE/MVB as a signaling hub as well as their content, e.g., CD63^+^ intralumenal vesicles (exosome precursors), could provide further insight on the role of migrasomes as communicative devices.

The future challenges will demonstrate whether MSC-derived migrasomes have an impact on the biology of hematopoietic stem cells, notably their fate and to explore the in vivo relevance of migrasomes in the bone marrow. Reciprocally, it will be interesting to determine whether microenvironmental conditions observed in cancers, such as hypoxia and low extracellular pH, or other diseases have an impact on migrasome formation by MSCs. Similarly, it will be of interest to evaluate MSCs derived from aged donors for their capacity to produce such specific organelle [63]. Finally, it remains to be evaluated whether MSC-associated migrasomes have therapeutic uses, like MSCs, in host tissues upon transplantation, such as immunomodulatory properties among others.

## Supplementary Information

**Additional file 1:** Supplemental Tables S1–S2 and Figures S1–S8.

**Additional file 2: Supplemental Video S1.** Phase contrast time-lapse video depicting the membrane retraction-based migrasome production mechanism by an MSC. The elapsed time is shown on the right corner. Still images from this video are shown in Fig. 1I. (Format, MOV; size, 5.3 Mb).

**Additional file 3: Supplemental Video S2.** Phase contrast time-lapse video depicting the migration-based migrasome production mechanism by an MSC. The elapsed time is shown on the right corner. Still images from this video are shown in Fig. 1J. (Format, MOV; size, 4.8 Mb).

**Additional file 4: Supplemental Video S3.** A 3D rendering of a migrasome from PFA-fixed and saponin-permeabilized cell immunolabeled with anti-α-tubulin antibodies (red) and stained by SiR-Actin (green) and fluorophore-conjugated WGA (white). Slicing through the z-levels shows the distribution of cytoskeleton in the migrasome and retraction fibers. Still images from this video are shown in Fig. 3B. (Format, MOV; size, 3.7 Mb).

**Additional file 5: Supplemental Video S4.** A time-lapse video of a cell stained with SiR-Actin (white) and imaged with total internal reflection fluorescence (TIRF) microscopy at a layer height of 250 nm. The elapsed time in minutes is shown on the right corner. White arrowhead points to movement of actin filaments in a retraction fiber. Still images from this video are shown in Fig. 3E. (Format, MOV; size, 2.9 Mb).

**Additional file 6: Supplemental Video S5.** A 3D rendering of a migrasome from a PFA-fixed and saponin-permeabilized cell immunolabeled for CD63 (red) and stained with fluorophore-conjugated WGA (green). Slicing through the z-levels shows the CD63^+^ vesicles within a migrasome. Still images from this video are shown in Fig. 4H. (Format, MOV; size, 3.7 Mb).

**Additional file 7: Supplemental Video S6.** A 3D rendering of a migrasome from a PFA-fixed and saponin-permeabilized cell expressing Rab7-GFP (green), immunolabeled for CD63 (red) and stained with fluorophore-conjugated WGA (green). Slicing through the z-levels shows the Rab7^+^ structures within the migrasome. Still images from this video are shown in Fig. 4L. (Format, MP4; size, 2.2 Mb).

**Additional file 8: Supplemental Video S7.** A 3D rendering of migrasomes from a transiently SDF-1-GFP (green) expressing, PFA-fixed cell were stained with fluorophore-conjugated WGA (white). Orbiting around the migrasomes shows the SDF-1^+^ vesicles within the migrasomes. Still images from this video are shown in Additional file 1: Fig. S4. (Format, MOV; size, 8.2 Mb).

**Additional file 9: Supplemental Video S8.** Phase contrast time-lapse video of MSCs with a migrasome network showing the detachment of migrasomes or release of their contents. The elapsed time is shown on the right corner. Still images from this video are shown in Fig. 5C. (Format, MOV; size, 6.2 Mb).

**Additional file 10: Supplemental Video S9.** Phase contrast time-lapse video of MSCs co-cultured with KG-1a cells, depicting the migration of KG-1a cells towards the MSC-associated migrasome network and their subsequent adhesion to retraction fibers. The elapsed time is shown on the right corner. Still images from this video are shown in Fig. 5H. (Format, MOV; size, 2.8 Mb).

**Additional file 11: Supplemental Video S10.** Phase contrast time-lapse video of MSCs co-cultured with KG-1a cells, depicting the migration of KG-1a cells towards the MSC-associated migrasome network and the absorption of migrasomes (white asterisks). The elapsed time is shown on the right corner. Still images from this video are shown in Fig. 5K. (Format, MOV; size, 7.7 Mb).

**Additional file 12: Supplemental Video S11.** Phase contrast time-lapse video of MSCs co-cultured with KG-1a cells, depicting the migration of KG-1a cells on the MSC-associated migrasome network and the absorption of cell-free detached migrasomes (white asterisk). The elapsed time is shown on the right corner. Still images from this video are shown in Additional file 1: Fig. S7. (Format, MOV; size, 4.4 Mb).

**Additional file 13: Supplemental Video S12.** Phase contrast time-lapse video of MSCs co-cultured with CD34^+^ HSPCs, depicting the migration of hematopoietic cells over the MSC-associated migrasomes (red asterisks) without absorbing them. The elapsed time is shown on the right corner. Still images from this video are shown in Additional file 1: Fig. S8. (Format, MOV; size, 9.6 Mb).

## Data Availability

Not applicable.

## Abbreviations

BSA: Bovine serum albumin
Col: Collagen
CCCP: Carbonyl cyanide m-chlorophenyl hydrazine
CLSM: Confocal laser scanning microscopy
CXCL12: C-X-C motif chemokine 12
CXCR4: C-X-C chemokine receptor 4
DMSO: dimethyl sulfoxide
ECM: Extracellular matrix
HSPCs: Hematopoietic stem and progenitor cells
LE/MVB: late endosomes/multivesicular bodies
MACS: Magnetic-activated cell sorting
MSCs: Mesenchymal stromal cells
PFA: Paraformaldehyde
PLL: Poly-l-lysine
S.D.: Standard deviation
SDF-1: Stromal cell-derived factor 1
SEM: Scanning electron microscopy
TIRF: Total internal reflection fluorescence
WGA: Wheat germ agglutinin
