# Supplementary material for: Mesenchymal stromal cell-associated migrasomes: a new source of chemoattractant for cells of hematopoietic origin
Source: Cell Commun Signal. 2023 Feb 14;21:36. doi: 10.1186/s12964-022-01028-6 (PMC9926842; doi:10.1186/s12964-022-01028-6)
Supplement: Supplementary file 2 — Additional file 1: Supplemental Tables S1–S2 and Figures S1–S8. [file 12964_2022_1028_MOESM2_ESM.docx]

**Supplementary Information**

**Additional file 1**

**Mesenchymal stromal cell-associated migrasomes – A new source of chemoattractant**

**for cells of hematopoietic origin**

Ilker A. Deniz, Jana Karbanová, Manja Wobus, Martin Bornhäuser, Pauline Wimberger, Jan Dominik Kuhlmann and Denis Corbeil

**Supplemental Figure S1-S8**

**Supplemental Tables S1 and S2**

**Supplemental Figures**

**
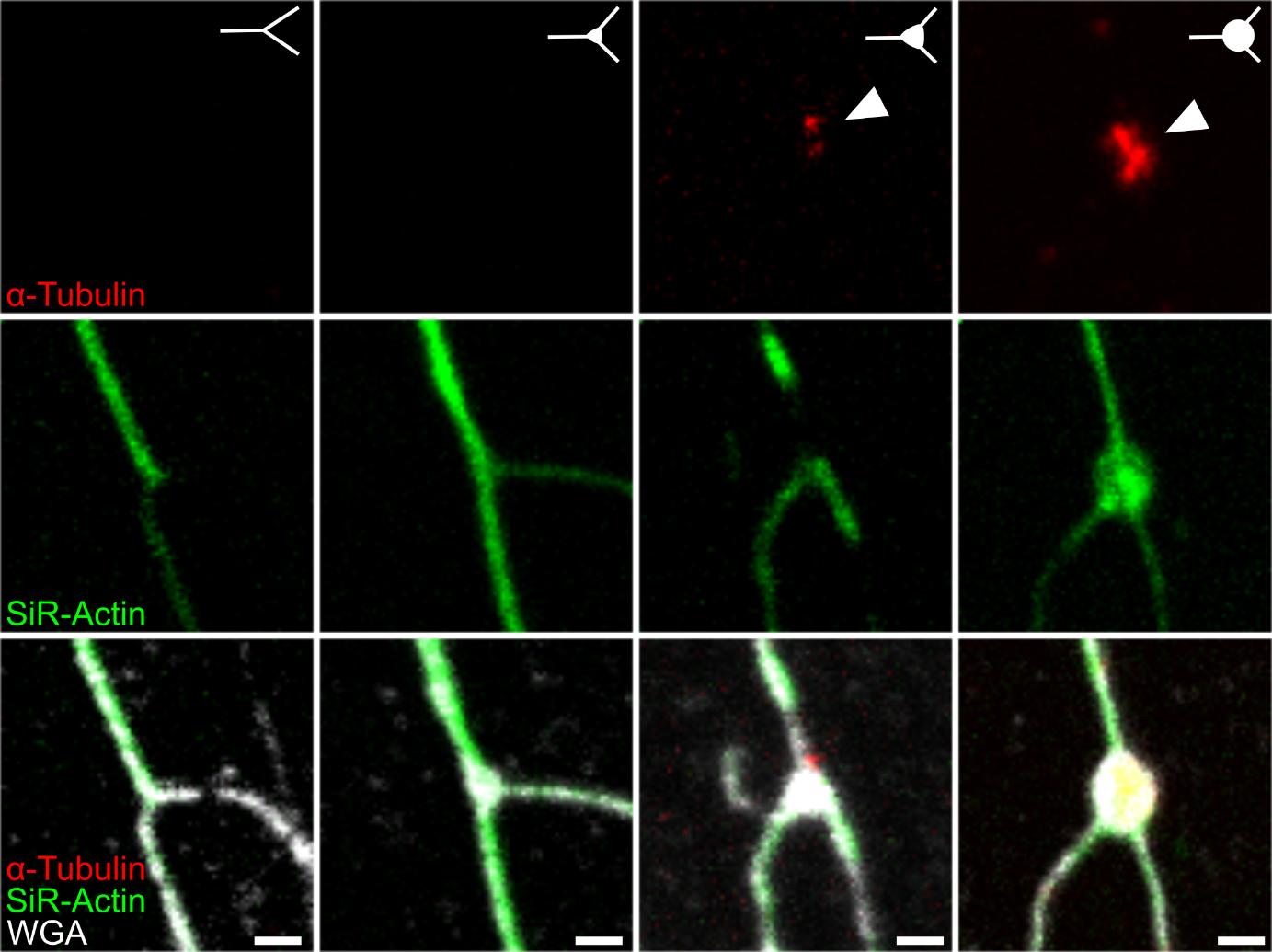
**

### Supplemental Figure S1.

**Distribution of α-tubulin changes during migrasome maturation.** Primary human MSCs were cultured on fibronectin-coated glass coverslips for 24 hours before being processed for CLSM. PFA-fixed cells were saponin-permeabilized prior to immunolabeling with α-tubulin antibodies, followed by staining by SiR-Actin and fluorophore-conjugated WGA, to label actin cytoskeleton and cellular membranes, respectively. Note that α-tubulin is absent in retraction fibers and immature migrasomes (first and second columns), while its amount increases throughout the maturation of migrasomes (third and four columns, arrowheads). These images are also presented in Fig. 3C. Scale bars, 1 µm.


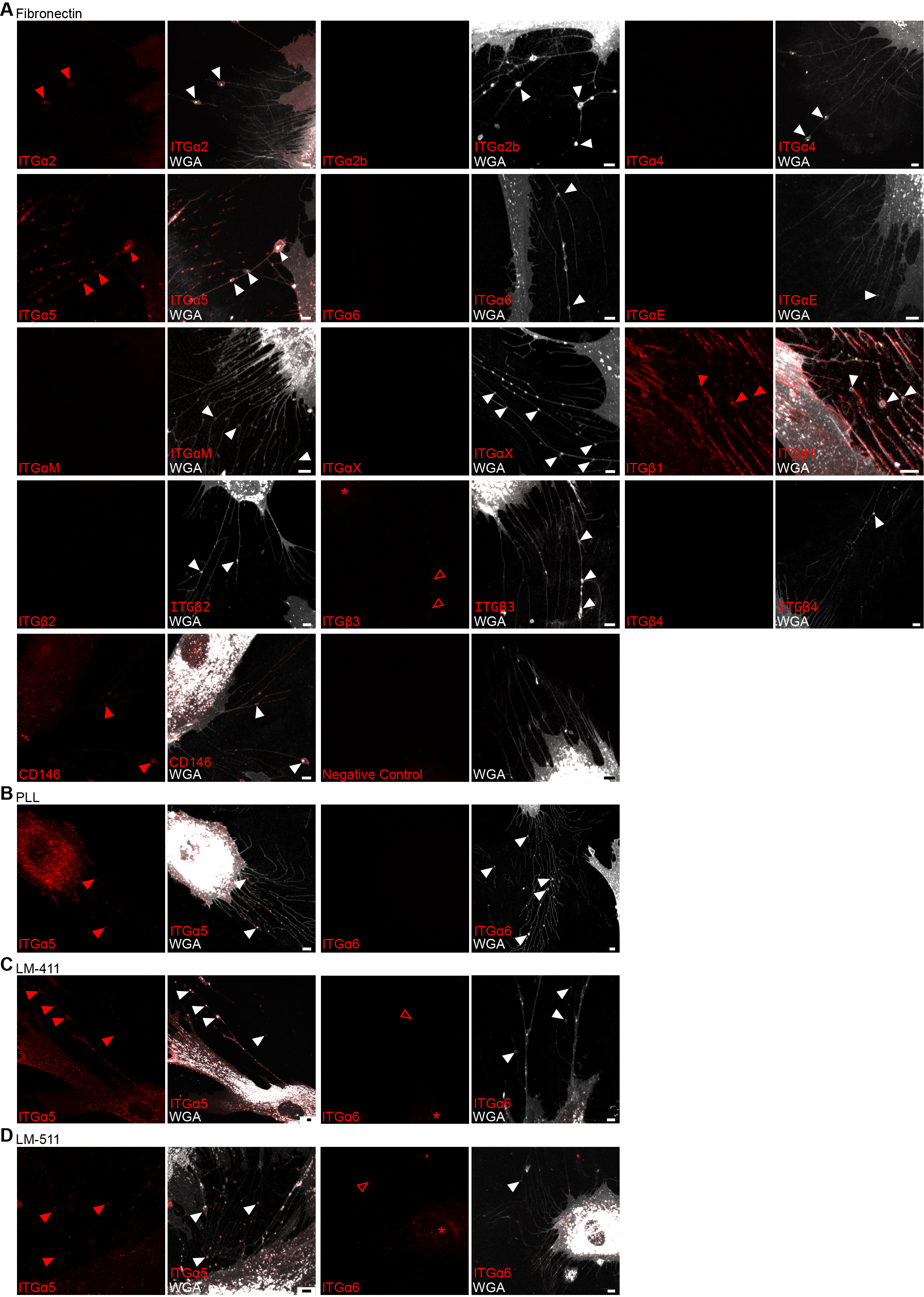


### Supplemental Figure S2.

**MSCs and associated migrasomes exhibit a distinct integrin profile.** (A-D) Primary human MSCs were cultured on fibronectin-(A), Poly-l-lysine- (B), LM-411- (C) or LM-511 (D) coated glass coverslips for 24 hours before being processed for CLSM. Cells were PFA-fixed followed by membrane permeabilization with saponin. Cells were immunolabeled with primary antibodies directed against specific integrin (ITG) or CD146 as indicated followed with the appropriate fluorophore-conjugated secondary antibody and by membrane staining with fluorophore-conjugated WGA. For negative control, samples were only stained with secondary antibody. White arrowheads indicate WGA-stained migrasomes, while red arrowheads indicate those positive for a given protein of interest. Open red arrowheads and asterisks show the very weak labeling of ITGβ3 or ITGα6 in migrasomes or cell body, respectively. Scale bars, 5 µm.


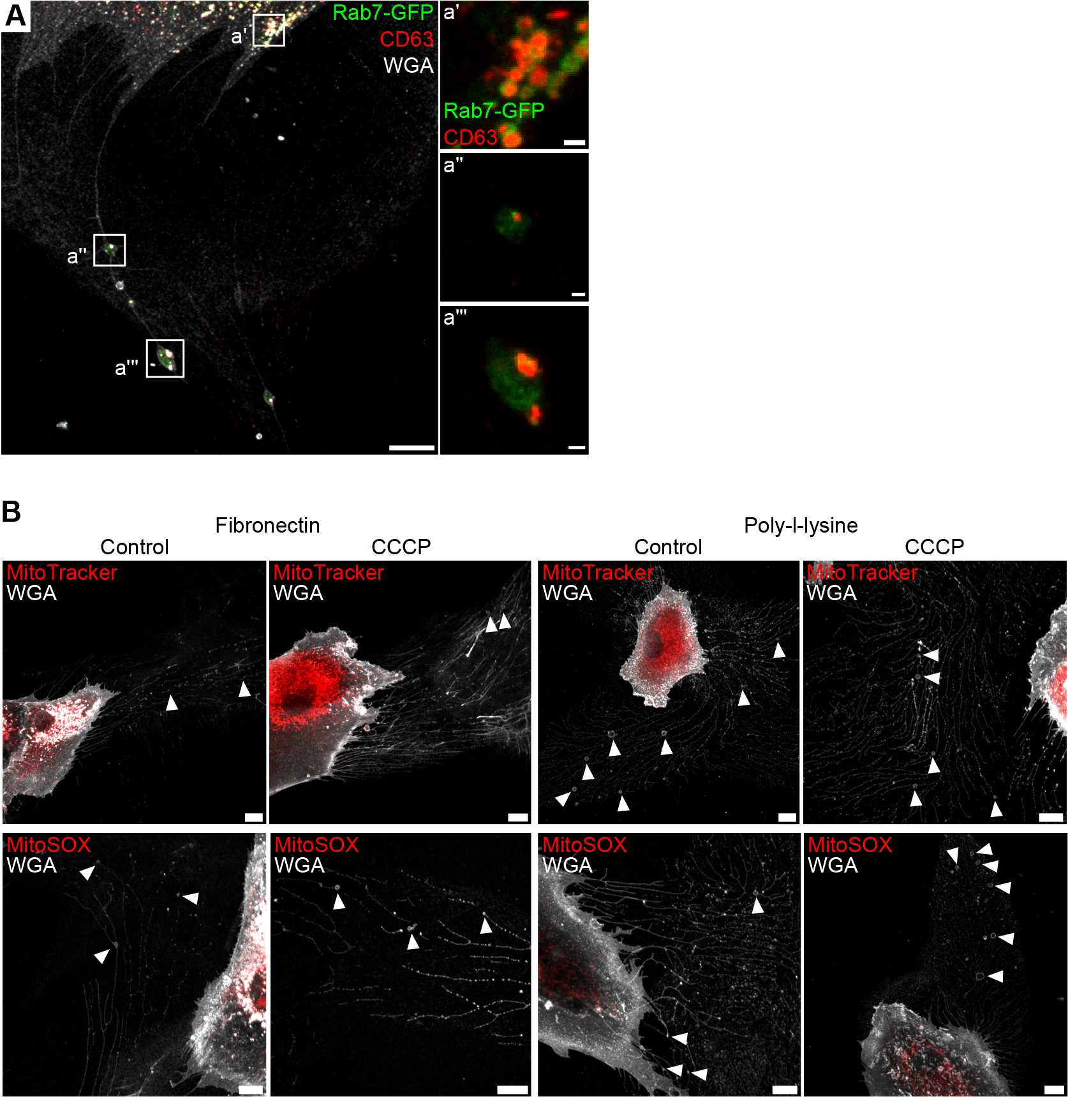


**Supplemental Figure S3.**

**Migrasomes contain late endosomes, but not mitochondria.** (A, B) Transiently Rab7-GFP–transfected MSCs (A) or non-transfected MSCs (B) were cultured on fibronectin- (A, B) or Poly-l-lysine- (B) coated glass coverslips for 24 hours before either, imaging (A), or treatment with DMSO as vehicle control (Control) or CCCP (2 µM), a mitochondrial uncoupler, for 8 hours followed by mitochondria staining using either MitoTracker or Mito-SOX dye (B). Cells were PFA-fixed, saponin-permeabilized before immunolabeling for CD63 (A) followed by fluorophore-conjugated WGA staining (A, B) before being processed for CLSM analysis. Note the co-localization/proximity of Rab7-GFP and CD63 in the cell (A, a') and migrasomes (A, a" and a"'), and the absence of mitochondria in WGA-stained migrasomes (B, arrowhead) compared to the cell body (B) under native and stress conditions (B). Scale bars, 10 µm (A, B), 1 µm (a’-a’”).


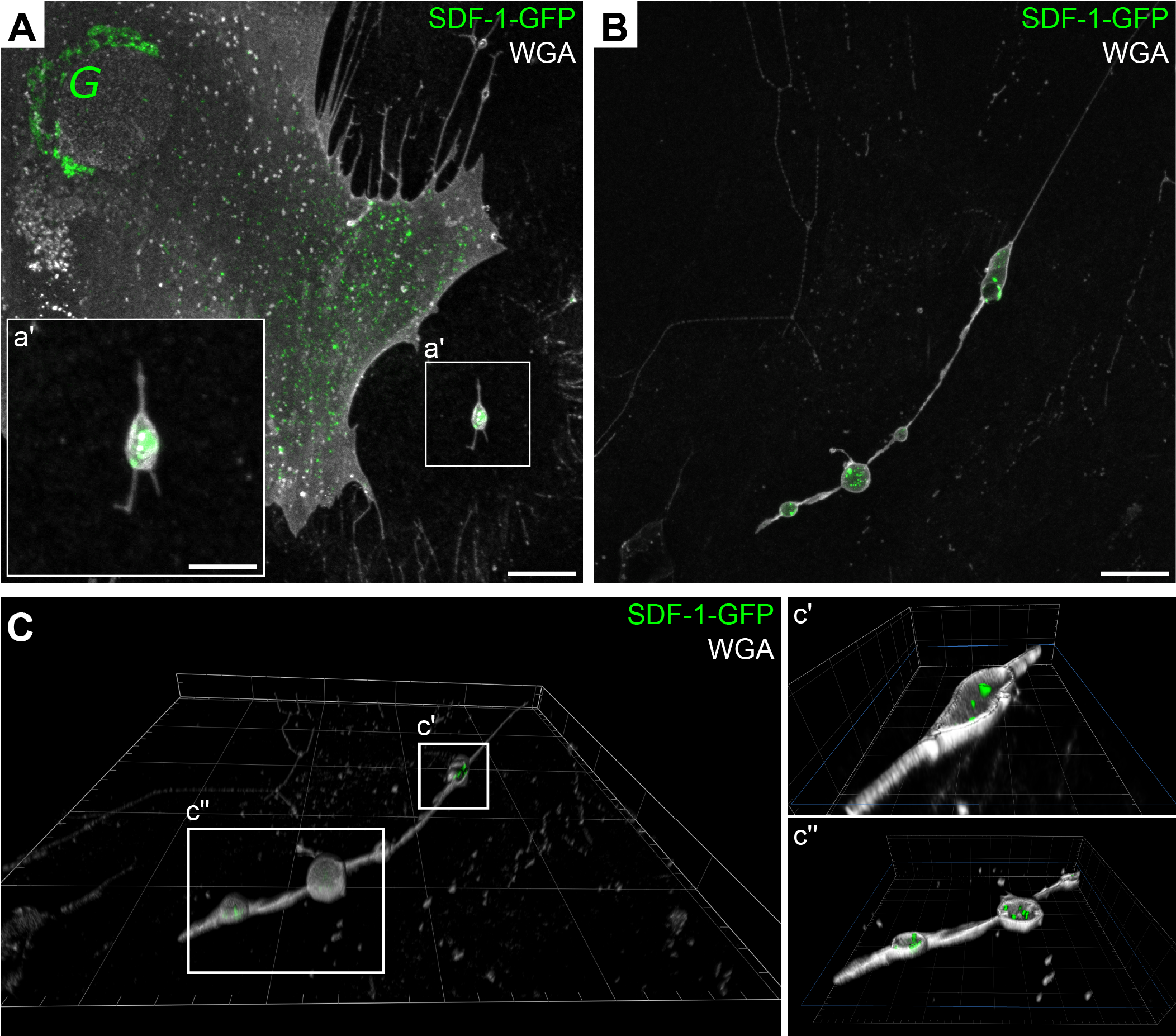


### Supplemental Figure S4.

**Cell-free migrasomes contain SDF-1.** (A-C) Transiently SDF-1-GFP–transfected MSCs were cultured on fibronectin-coated glass coverslips for 24 hours before PFA-fixation and staining with fluorophore-conjugated WGA. The presence of SDF-1 can be observed throughout the cell cytoplasm (A) with its accumulation in the Golgi apparatus (*G*) as well as in cell-free detached migrasomes (A, inset a’) or those remaining attached to the remnant retraction fibers (B). A 3D render of migrasomes presented in panel B highlights the presence of SDF-1-GFP inside them (C’, insets c’ and c’’). The image is excerpted from the Supplemental Video S7. Scale bars, 10 µm (A, B), 1 µm (a’).

**
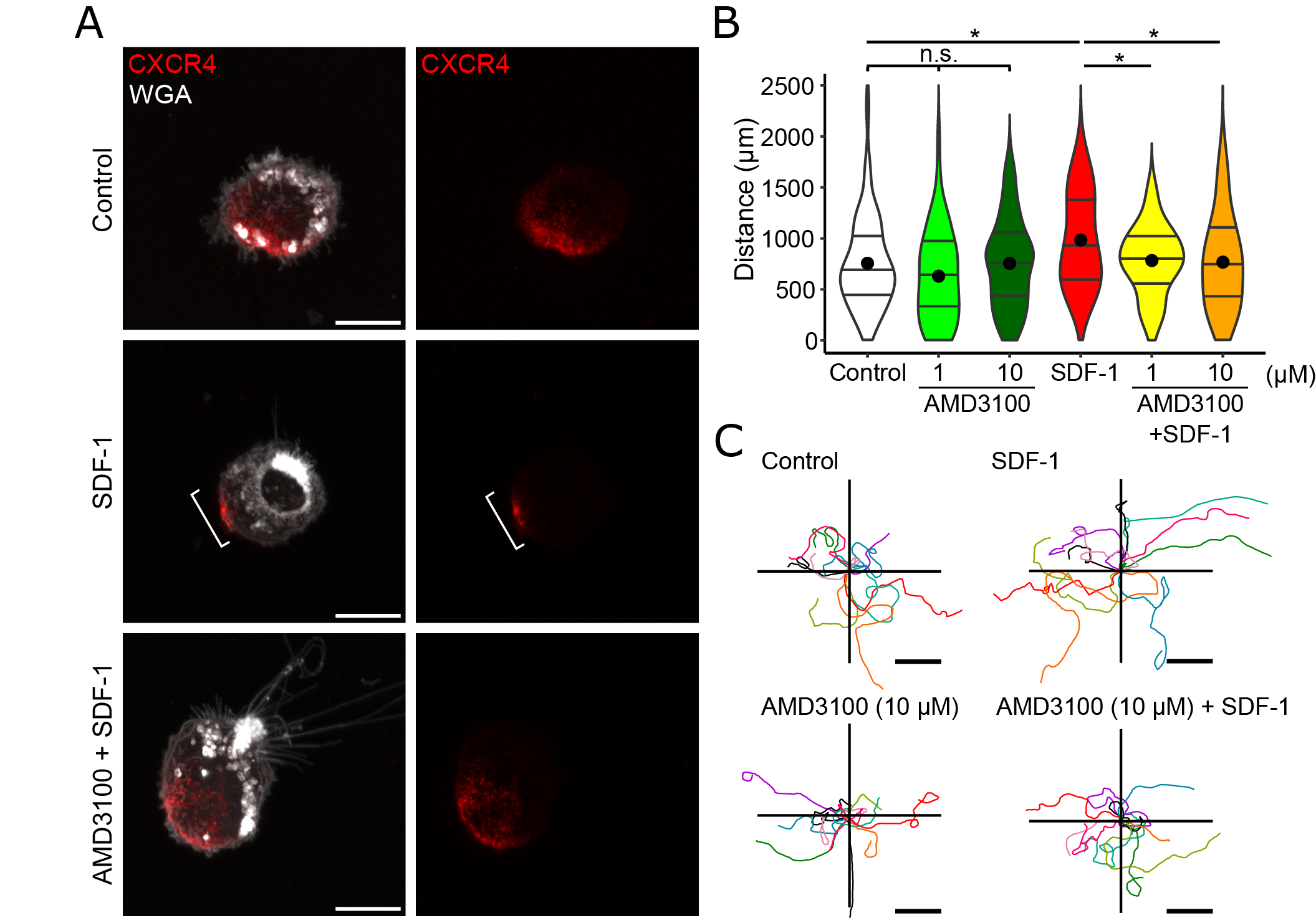
**

### **Supplemental Figure S5**.

**CXCR4 localizes to leading edge upon activation by SDF-1.** (A-C) KG-1a cells were seeded on fibronectin-coated glass coverslips in the presence of either AMD3100 (1 or 10 µM) or SDF-1 (100 ng/mL) or a combination of both for 8 hours before PFA fixation and CLSM processing (A) or recording by live-cell phase contrast microscopy (B-C). Fixed cells were immunolabeled for CXCR4, followed by fluorophore-conjugated WGA staining (A). Note the concentration of CXCR4 at the leading edge of the cell membrane upon SDF-1 stimulation (bracket), while the combination of SDF-1 and AMD3100 shows a more distributed localization as the control. Live cell imaging was performed at 15-minute intervals and the migration of individual cells are quantified and plotted (B, C). The dots in violin plots represent the mean distance, while the horizontal lines represent the 25^th^ percentile, median and 75^th^ percentile, from bottom to top, respectively (B, >100 cells were quantified). Medians of each population were compared using Mann-Whitney U test. Representative migration tracks of 10 individual cells are shown for the conditions indicated (C). N.s., not significant, * *p* < 0.05. Scale bars, 10 µm (A), 250 µm (C).

**
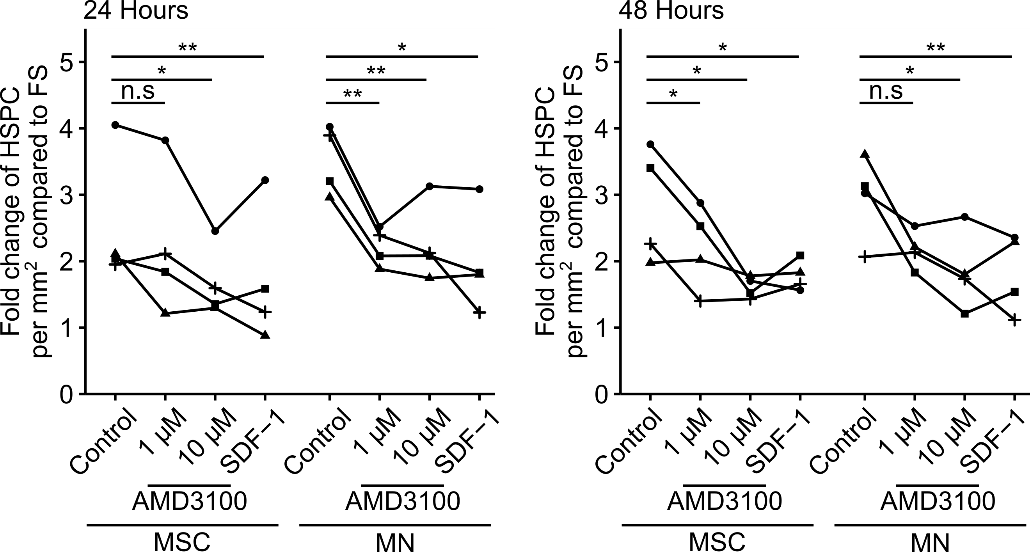
**

### Supplemental Figure S6.

**CD34^+^ HSPCs are attracted to MSC-derived migrasomes in a CXCR4–SDF-1 axis dependent manner.** 24 or 48 hours after their immunoisolation, primary CD34^+^ HSPCs were co-cultured with sub-confluent MSCs in the absence (control) or presence of either 1 or 10 µM AMD3100 or 100 ng/mL recombinant SDF-1 for a period of 4 hours. Afterward, cells were live-imaged and the distribution of hematopoietic cells on MSCs (MSC), the migrasome network (MN) and free surface (FS) was quantified. Data are presented as the fold change in the density of CD34^+^ HSPCs per mm^2^ by comparison to FS. Symbols indicate four individual donors. At least 200 cells were evaluated per condition. Mean of experiments were compared against control using students T-test paired for individual donors. N.s., not significant, * *p* < 0.05; ** *p* <0.01.

###
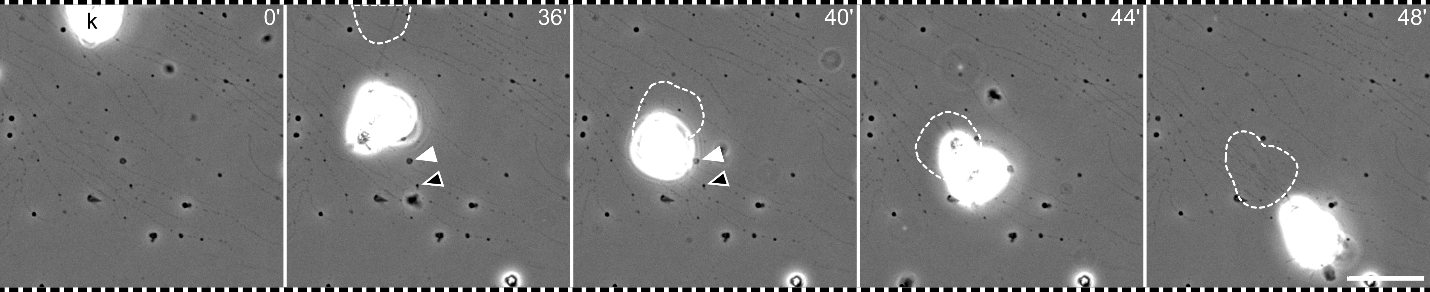


### Supplemental Figure S7. Migrating KG-1a cells absorb cell-free migrasomes. Primary human MSCs cultured on fibronectin-coated glass coverslips for 24 hours before addition of KG-1a cells (k) followed by imaging by live-cell phase-contrast microscopy for a 12-hour period. Dashed lines show the position of the leukemic cell in the previous frame, while the arrowheads show cell-free detached MSC-derived migrasomes that are absorbed or taken up by migrating leukemic cell. The elapsed time in minutes is shown on the top right corner. All frames are excerpted from Supplemental Video S11. Scale bar, 10 µm.


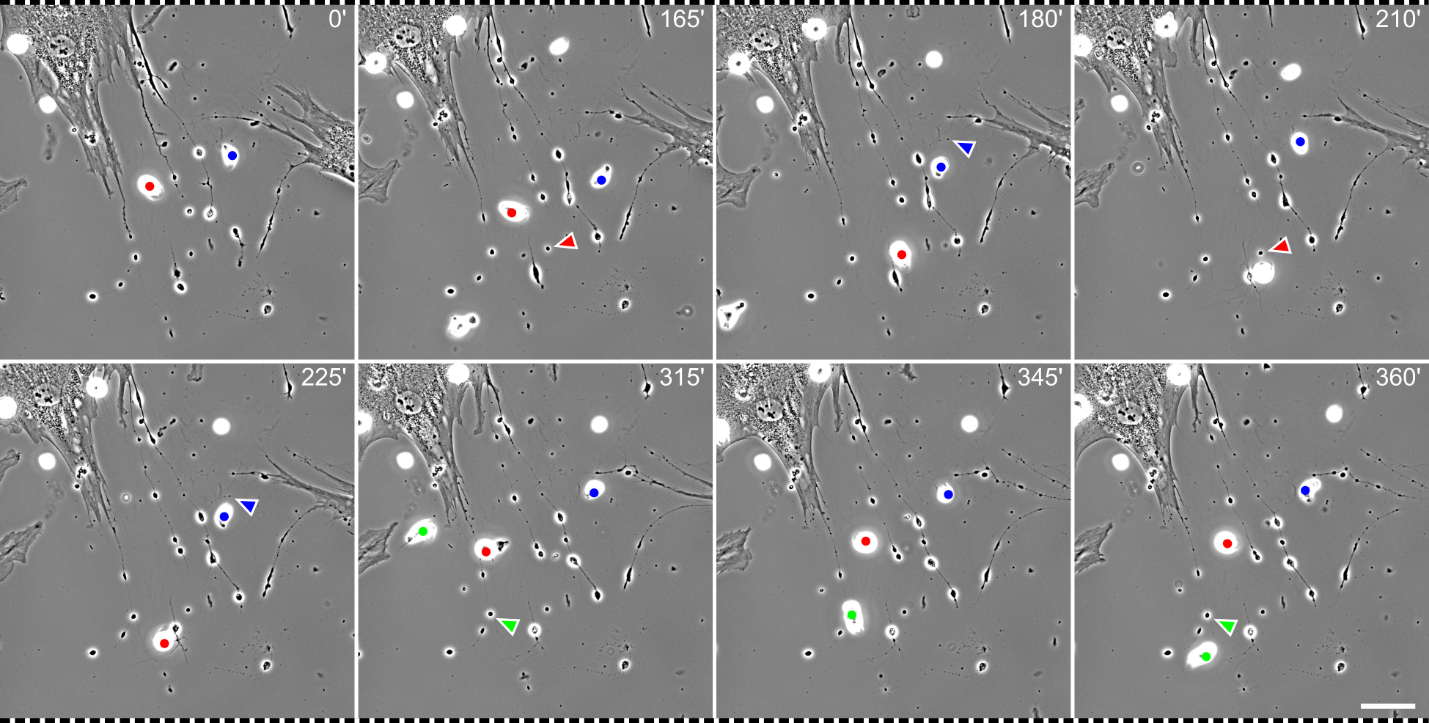


### Supplemental Figure S8.

**Migrating CD34^+^ HSPCs do not absorb MSC-associated migrasomes.** Primary human MSCs were cultured on fibronectin-coated glass coverslips for 24 hours before addition of CD34^+^ HSPCs (colored solid circles) followed by imaging using live-cell phase-contrast microscopy for a 12-hour period. Three-colored arrowheads indicate MSC-associated migrasomes that are encountered by hematopoietic cells without being taken up. The elapsed time in minutes is shown on the top right corner. All frames are excerpted from Supplemental Video S12. Scale bar, 50 µm.

# Supplementary Tables

## Table S1. List of primary antibodies

| **Primary antibody** | **Clone** | **Manufacturer**^1^ | **Dilution for immunofluorescence** |
| --- | --- | --- | --- |
| α-tubulin | DM1A | Sigma-Aldrich | 1:500 |
| CD9 | HI9a | BioLegend | 1:100 |
| CD11b-PE^*, 2^ | M1/70.15.11.5 | Miltenyi Biotec | 1:50 |
| CD11c-APC^*^ | B-ly6 | BD Biosciences | 1:50 |
| CD18 | 6.7 | BD Biosciences | 1:50 |
| CD41a | HIP8 | BD Biosciences | 1:50 |
| CD44 | DF1485 | Santa Cruz Biotech. | 1:100 |
| CD49b-APC^*^ | P1E6-C5 | BioLegend | 1:50 |
| CD49d-APC^*^ | MZ18-24A9 | Miltenyi Biotec | 1:50 |
| CD49e-APC^*^ | NKI-SAM-1 | BioLegend | 1:50 |
| CD49f^2^ | GoH3 | BD Biosciences | 1:50 |
| CD61-APC^*^ | VI-PL2 | Biolegend | 1:50 |
| CD63 | MX-49.129.5 | Santa Cruz Biotech. | 1:500 |
| CD73 | AD2 | BD Biosciences | 1:50 |
| CD81 | M38 | Exbio | 1:75 |
| CD90 | OX-7 | Acris antibodies | 1:50 |
| CD103-APC^*^ | B-ly7 | Thermo-Fisher | 1:50 |
| CD104 | 58XB4 | Biolegend | 1:50 |
| CD105 | SN6 | Invitrogen | 1:50 |
| CD146-PE^*^ | TEA 1/34 | Beckman-Coulter | 1:50 |
| CD166 | 3A6 | BD Biosciences | 1:200 or 5 µg/mL^4^ |
| Tspan2^3^ | – | Thermo-Fisher | 1:200 |
| Integrin beta-1 | 12G10 | Abcam | 1:50 |
| Tspan4^3^ | – | Novus biologicals | 1:200 |
| Rab7^3^ | D95F2 | Cell signaling | 1:200 |
| SDF-1 | 79018 | R&D systems | 1:30 |
| CXCR4 | 12G5 | BD Biosciences | 1:50 |
| IgG1 Isotype control | MOPC 21 | Sigma-Aldrich | 5 µg/mL^4^ |

^1^Abcam (Cambridge, UK); Acris antibodies (Bielefeld, Germany); BD Biosciences (Heidelberg, Germany); Beckman-Coulter (Krefeld, Germany); BioLegend (San Diego, CA, USA); Cell Signaling Technology (Danvers, MA, USA); Exbio (Prague, Czech Republic); Miltenyi Biotec (Bergisch Gladbach, Germany); Novus Biologicals (Littleton, CO, USA); R&D systems (Minneapolis, MN, USA); Santa Cruz Biotechnology, Inc. (Dallas, TX, USA); Sigma Aldrich (Darmstadt, Germany); Thermo Fisher Scientific (Waltham, MA, USA)

^2^Antibody generated in rat

^3^Antibody generated in rabbit

^4^ Concentration used to block cell surface CD166 on MSCs and migrasomes

*Note that PE (phycoerythrin) and APC (allophycocyanin) were not used for integrin immunodetection

## Table S2. List of secondary antibodies

| **Secondary antibody** | **Manufacturer** | **Dilution for immunofluorescence** |
| --- | --- | --- |
| Alexa Fluor™ 488–conjugated goat anti-mouse IgG (H+L) | Thermo Fisher Scientific^1^ | 1:400 |
| Alexa Fluor™ 546–conjugated goat anti-mouse IgG (H+L) | Thermo Fisher Scientific | 1:400 |
| Alexa Fluor™ 488–conjugated goat anti-rabbit IgG (H+L) | Thermo Fisher Scientific | 1:400 |
| Alexa Fluor™ 546–conjugated goat anti-rat IgG (H+L) | Thermo Fisher Scientific | 1:400 |
| Alexa Fluor™ 546–conjugated goat anti-mouse IgG2a (H+L) | Thermo Fisher Scientific | 1:400 |

**^1^**Thermo Fisher Scientific (Waltham, MA, USA)
